# Heat Stress Impairs the Physiological Responses and Regulates Genes Coding for Extracellular Exosomal Proteins in Rat

**DOI:** 10.3390/genes11030306

**Published:** 2020-03-13

**Authors:** Jinhuan Dou, Adnan Khan, Muhammad Zahoor Khan, Siyuan Mi, Yajing Wang, Ying Yu, Yachun Wang

**Affiliations:** 1 Key Laboratory of Animal Genetics, Breeding and Reproduction, MARA, National Engineering Laboratory for Animal Breeding, College of Animal Science and Technology, China Agricultural University, Beijing 100193, China; 2 State Key Laboratory of Animal Nutrition, Beijing Engineering Technology Research Centre of Raw Milk Quality and Safety Control, College of Animal Science and Technology, China Agricultural University, Beijing 100193, China; wangyajing_cau@163.com

**Keywords:** thermal regulation, transcriptome, blood, liver, adrenal glands, rat model, biomarker

## Abstract

Heat stress (HS) is challenging in humans and animals as it is a complicated regulatory mechanism. This prompted us to characterize the physiological and molecular responses of a HS-animal model. In this study, a rat model system was developed by using three temperature treatments (40 ℃, 42 ℃, and 43 ℃) and sixteen biochemical indicators in blood at 42 ℃ for 30 min (H30), 60 min (H60), and 120 min (H120). In addition, transcriptomic profiling was carried out in H120-rats’ blood, liver, and adrenal gland samples for detection of the genes of interest. Our findings demonstrated that the adrenocorticotropic hormone, catalase, prolactin, growth hormone, and lactic acid have significant spatiotemporal variation in the H120-rats as compared with the control. Furthermore, through transcriptomic screening, we documented a high ratio of differentially expressed genes (DEGs) in adrenal glands, liver, and blood, respectively. Among them, *Nup153*, *Plxnb2*, *Stx7*, *Hspa9*, *Chordc1*, *Pde4d*, *Gm2α,* and *Rnf125* were associated with the regulation of HS and immune response processes. Notably, 36 and 314 of DEGs in blood and adrenal glands were detected in the composition of the extracellular exosome, respectively. Furthermore, the correlation analysis between gene transcripts and biochemical indicator levels identified the *Lgals3, S1006, Fn1,* *F2,* and *Kng1l1* as key candidate genes for HS encoding extracellular exosomal proteins. On the basis of our results, it was concluded that the current rat model provides a molecular basis for future research in HS resistance in humans and livestock.

## 1. Introduction

The negative influence of a hot climate on humans and animals is challenging not only in tropical and subtropical areas, but also in temperate regions [1,2]. In humans, long-term exposure to high environmental temperature can induce heatstroke or heat stress (HS) and can even lead to increased morbidity and mortality [3,4]. Heat stress is a nonspecific body response resulting from the pathological increase in core body temperature without an increase in the temperature set point [5]. In livestock, HS can lead to a decrease in fertility, as well as reduced milk and meat production resulting in great economic losses [6,7,8]. Management measures for preventing HS, such as improvement of housing conditions, application of different nutritional regimens, or breeding heat tolerant breeds (e.g., crossbreeding), have not achieved long-lasting, cumulative and significant effects [9]. Thus, further molecular mechanisms and physiological consequences of HS using an animal model are highly recommended.

Previous studies have shown HS-induced physiological and biochemical variations in many animals, such as rats [10,11], cows [12], and chickens [13]. The HS causes an imbalance of glucocorticoids, the adrenocorticotropic hormone, the growth hormone, and the norepinephrine hormone, resulting in detrimental metabolic changes [10,14,15]. Furthermore, a set of proteins involved in the antioxidant stress response and inflammatory response, including catalase, glutathione peroxidase, and C-reactive protein were affected by HS [16,17,18,19]. Recently, microarray and next-generation sequencing studies have displayed widespread changes in gene expression in response to HS, however [20,21,22,23] the exploration of useful indicators to predict and prevent HS in animals has yet to be defined. 

In the context of the critical effects of HS on human and animal health, the current study was designed to explore the physiological, biochemical, and transcriptomic responses to HS in Sprague Dawley (SD) rats. The current research targets the key biomarkers that are sensitive to HS. In addition, the altered expression of genes related to HS response was detected by performing transcriptomic screening in blood, liver, and adrenal gland tissues post HS. Collectively, our data provides new insight into the molecular consequences of the rising global temperatures for both humans and livestock. 

## 2. Materials and Methods 

### 2.1. Ethics Approval

All procedures and protocols for the experimental rats were approved by the Institutional Animal Care and Use Committee at the China Agricultural University, China under permission number DK996. The *in Vivo* rat experiments were performed at the College of Animal Science and Technology, China Agricultural University. The Institutional Animal Care and Use Committee approved all the experimental procedures, which complied with the China Physiological Society’s guiding principles for research involving animals and adhered to the high standard (best practice) of veterinary care as stipulated in the Guide for Care and Use of Laboratory Animals. 

### 2.2. Animal Model and Treatments

As previously described [11], 8-week-old female specific-pathogen-free (SPF) SD rats (Beijing Vital River Laboratory Animal Technology Co., Ltd., Beijing, China) weighing 205 ± 7.16 g were used as subjects. Prior to the HS experiments, a total of three rats per cage were housed in Nalgene polycarbonate cages (40 × 30 × 180 cm^3^, Beijing Vital River Laboratory, Animal Technology Co, Ltd., Beijing, China) at 22 ± 1 °C (room temperature) with a 12 h reverse light/dark cycle (on 06:00 h, off 18:00 h) for one week. The relative humidity (RH) for all experiments was controlled at 50%. We provided food and water *ad libitum* and all experiments were conducted with healthy and conscious rats.

**Figure 1:**
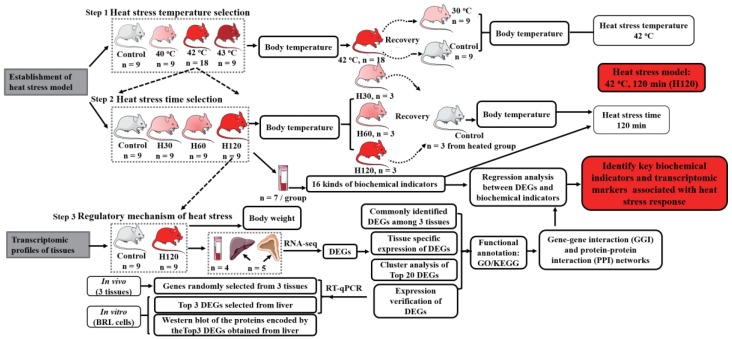
Experimental design of the study. First, heat stress (HS) conditions were explored by analyzing the body temperature of rats at different high ambient temperatures or treatment times, as well as body temperature recovery after transferring to 30 °C or 22 ± 1 °C from HS conditions (Steps 1 and 2). After 42 °C HS for 30 min (H30), 60 min (H60), and 120 min (H120), animals were euthanized, and their blood biochemical indicators were measured (Step 2). RNA-sequencing was used to establish the transcriptomic profiles of blood (*n* = 4), liver (*n* = 5), and adrenal gland (*n* = 5) tissues of rats in H120 and control groups (Step 3). Differentially expressed genes (DEGs) analysis, its validation, gene ontology terms (GO), and Kyoto Encyclopedia of Genes and Genomes (KEGG) pathway analysis, and gene–gene and protein–protein interaction network (GGI and PPI) construction were conducted to characterize the common and specific DEGs responses to HS in blood, liver, and adrenal glands. Finally, the Pearson correlation analysis of the DEGs in three tissues and the biochemical indicators in blood was performed.

Ninety-nine rats were used in two different experiments (Figure 1). Firstly, a HS model was established by comparing changes in physiological and biochemical indicators under different intensities (Step 1) and durations (Step 2) of HS. Forty-five experimental rats were randomly assigned to the following four treatment groups: the control (22 ± 1 °C, *n* = 9) and the heat treatments at 40 °C (*n* = 9), 42 °C (*n* = 18), or 43 °C (*n* = 9) (Step 1). The heating experiments were completed in a floor-standing artificial climate incubator (BIO250, BOXUN Medicine Instrument Co, Shanghai, China). Rats in the control group were never introduced to the incubator. An electronic clinical thermometer with a precision of ± 0.1 °C (MC-347, Omron Corporation, Kyoto, Japan) was used to detect the body temperature of rats from each group every hour for 24 h. After exposure to 42 °C for 24 h, rats were transferred to lower ambient temperature conditions, 30 °C (*n* = 9) or 22 ± 1 °C (*n* = 9), for recovery until reaching their normal body temperature. After selecting the HS temperature, another set of 36 rats were used in a HS timed selection experiment at 42 °C for 30 min (H30, *n* = 9), 60 min (H60, *n* = 9), or 120 min (H120, *n* = 18) (Step 2). The body temperature of rats under various HS durations was reported according to the previously published study [11]. In addition, sixteen biochemical indicators of blood in each group (*n* = 7/group) were measured, including dopamine, noradrenaline, epinephrine, adrenocorticotrophic hormone, growth hormone, prolactin, lipid peroxidase, C-reactive protein, lactic dehydrogenase, lactic acid, malonaldehyde, catalase, glutathione peroxidase, superoxide dismutase, sodium-potassium ATPase (NA^+^/K^+^-ATPase), and urea nitrogen. After exposure to 42 °C, rats in the H30, H60 and H120 groups were transferred to room temperature for body temperature recovery. Secondly, eighteen rats in the control (*n* = 9) or heat-treated (H120, *n* = 9) groups were used to investigate the transcriptomic regulation of HS (Step 3). Firstly, rats in the H120 groups were weighed before and after HS using an electronic scale (YP10002, Shanghai Youke Instrument Co., Ltd., Shanghai, China). Secondly, blood (*n* = 4), liver (*n* = 5), and adrenal glands (*n* = 5) from the same five rats in the control and H120 groups were used to identify the differentially expressed genes (DEGs).

### 2.3. Blood and Tissue Collection 

Rats in control, H30, H60, and H120 groups were anesthetized with 1.2 mL 1% pentobarbital sodium (40 mg/kg body weight). Approximately 4 mL of blood from each rat was collected, stored in vacuum blood collection tubes (Becton, Dickinson and Company, Franklin, MA, USA), and heparinized tubes (vacuum plasma collection tube, Becton, Dickinson and Company, Franklin, MA, USA), and immediately placed on ice. Serum in the vacuum blood collection tubes was separated via centrifugation (4 °C, 5 min, 3500 rpm) and stored at −20 °C until further analyses. The white film layer in the middle of the blood stored in heparinized tubes was collected by RNase-free spear after centrifugation (10 min, 3000 rpm/min), transported to a new 2 mL centrifuge tube containing 1 mL Trizol (Invitrogen 15596018, Thermo Fisher Scientific Inc., Waltham, MA, USA) and stored at −80 °C for RNA extraction. Liver and adrenal gland tissues were washed in ice-cold phosphate buffer solution (PBS) and snap-frozen immediately in liquid nitrogen until further analysis. 

### 2.4. Biochemical Indicators Determination and Analysis

The dopamine, noradrenaline, and epinephrine levels were detected using an enzyme-linked immunosorbent assay. The adrenocorticotrophic hormone, growth hormone, prolactin, lipid peroxidase, and C-reactive protein levels were measured using the radio immunological method. The lactic dehydrogenase, lactic acid, malonaldehyde, catalase, glutathione peroxidase, superoxide dismutase, sodium-potassium ATPase (NA^+^/K^+^-ATPase), and urea nitrogen levels were measured using the blood biochemical method [24,25,26]. Intra- and inter-assay coefficients of variation were calculated by analyzing standards in each assay. Intra-assay variation was 9.5%, and the inter-assay variation was 7.5%.

### 2.5. RNA Extraction and Quality Assessment

Total RNA was isolated from rat blood, liver, and adrenal gland tissues for the control and H120 group. A white film cell layer (~150 μL) was mixed with 1 mL Trizol 20 times at 4 °C for 5 min to digest the cells. Samples were ground for 5 min at 35 Hz using a lapping machine (Scietz-48, Ning Bo, China), and stewed for 5 min for further processing. Next, RNA isolation was performed using thee chloroform extraction procedure. Frozen liver and adrenal glands stored in liquid nitrogen were placed on ice and about 100 mg tissues were cut into aliquots with a sterile scalpel and placed in new tubes containing 1 mL Trizol on dry ice. The RNA extraction procedure for blood followed the sample protocol. The quantity of RNA samples was evaluated using the NanoDrop 2000 (Thermo Fisher Scientific, Waltham, MA, USA). The RNA integrity was assessed using the RNA Nano 6000 Assay Kit in the Agilent Bioanalyzer 2100 system (Agilent Technologies, Santa Clara, CA, USA). All samples showed a 260/280 ratio changing range from 1.8 to 2.0, and integrity numbers (RIN) > 7.0.

### 2.6. Transcriptome Library Construction And Paired-End Sequencing

A total of 3 μg RNA per sample was used for RNA-sequencing (RNA-seq) library construction. Sequencing libraries were generated using NEBNext^®^ UltraTM Directional RNA Library Prep Kit from Illumina^®^ (NEB, San Diego, CA, USA) following the manufacturer’s protocol. Briefly, mRNA was purified and enriched from total RNA using Poly-T oligo-attached magnetic beads. Purified mRNA was fragmented using divalent cations under elevated temperature in NEBNext First Strand Synthesis Reaction Buffer (5×). First-strand cDNA was generated using random hexamer primers and M-MuLV Reverse Transcriptase (RNase H). Second strand cDNA was synthesized using DNA polymerase I and RNase H. The library fragments were purified with AMPure XP system (Beckman Coulter, Beverly, MA, USA) to select cDNA fragments approximately 200 bp in length, and then PCR amplified. Finally, the library was sequenced in paired-end 150 bp reads using Illumina^®^ HiSeq 2000 platform. The sequence data has been submitted to the NCBI/SRA database under accession number SUB6546585.

### 2.7. Assembly of RNA Reads and Identification of DEGs

Raw reads in the FASTQ format were processed using in-house Perl scripts removing adapter sequences, reads with more than 10 unknown nucleotides and low-quality reads (i.e., more than 50% of the reads with a quality score of under 10 or read length < 30). Quality control was conducted via FastQC [27] and Q20, Q30 and GC content were calculated. 

Bowtie2 [28] was used to index the rat reference genome (Rattus norvegicus 6.0) [29]. The TopHat2 [30] default parameter setting was applied to align the paired-end reads to the reference genome. Only reads uniquely aligned to the reference genome were used for downstream analysis. Then, Cufflinks [31] was used to assemble the aligned reads and the Cuffdiff function was applied to identify DEGs between control and H120 groups. The FPKM (fragments per kilobase per million mapped fragments) normalization procedure was used to account for different gene lengths and different sequencing depths across RNA-seq libraries. 

### 2.8. Bioinformatic Analysis of DEGs

Pearson correlation coefficient between and within control and H120 groups were calculated using the corrplot function in the package of R v3.5.0 software (TUNA team, Beijing, China). The false discovery rate (FDR), termed as *Q*-values, was calculated for differential expression analysis. Genes with *q* < 0.05 were considered as DEGs. The hierarchical clustering analysis of expression profiles of the DEGs in blood, liver, and the adrenal glands was performed using Pheatmap package in R v3.5.0 software.

The DEGs with *q* < 0.05 and absolute log2 (fold change) >1 were used for further analysis. The functional enrichment analysis of DEGs considering gene ontology terms (GO) and Kyoto Encyclopedia of Genes and Genomes (KEGG) pathway was conducted using KOBAS 3.0 [32]. The functional enrichment results of DEGs with *q* < 0.05 were considered as significant. The gene–gene interaction (GGI) and protein–protein interaction (PPI) networks were established using Gene MANIA [33] and STRING [34]. According to previous studies [33,35], the GGI network was constructed based on six types of evidence (co-expression, colocalization, pathway, shared protein domains, physical interaction and association network), the PPI network was examined based on four types of evidence (experimental, text mining, co-expression, and databases). 

### 2.9. RNA Reverse Transcription and Real-Time Quantitative PCR (RT-qPCR) Validation

The TransScript One-step g DNA Removal and cDNA Synthesis SuperMix Kit (Trans, Beijing, China) were used to synthesize cDNA with 2 µL of total RNA from each tissue. The relative expression of each target gene was normalized to that of glyceraldehyde-3-phosphate dehydrogenase (*GAPDH*). Gene-specific primers (*Cryab*, *Hspb1*, *Hspb8*, *Hsp90ab1*, *Hsf2*, *Nr3c1*) were cited from previous research [36,37,38,39,40,41], the primers of *Cdkn1a*, *Cryl1*, *Fabp1*, *Pck1* and *Sephs2* were designed using Primer-BLAST [42] and Oligo v7 (Molecular Biology Insights, Inc., Cascade, CO, USA), and their sequences are shown in Table S1. Gene expression was measured by real-time quantitative PCR (RT-qPCR) using SYBR green master mix (Applied Biosystems) following the manufacturer’s instructions. Triplicate RT-qPCRs were accomplished on each cDNA. RNA expression levels relative to the *GAPDH* gene were calculated as 2^−ΔΔ^Ct, according to previous research [43]. Fold change values were log_2_ transformed in order to compare the results from the RNA-seq analysis and RT-qPCR. 

### 2.10. The Buffalo Rat Liver (BRL) Cell Culture and HS Treatment for in Vitro Validation

Buffalo rat liver (BRL) cells were cultured in Dulbecco’s modified Eagle’s medium supplemented with 10% fetal bovine serum, 100 U/mL penicillin, and 100 µg/mL streptomycin in a humidified atmosphere of 5% CO_2_ at 37 °C. Cells were passaged using ethylene diaminetetraacetic acid (EDTA)-trypsin to reach 85% to 95% confluence, and after two passages, cells were exposed to H120. The control group was maintained at normal environmental conditions. Each experimental treatment was conducted in triplicate. Cell lysates were collected and stored at -80 °C for RNA and protein extraction. The RNA extraction and RT-qPCR for BRL cells *in vitro* validation experiments were conducted using the same protocol mentioned above. The sequence of *Hspa1b* mRNA was obtained from the NCBI GeneBank database (accessions NC_005119.3). Using this sequence, the primer was designed for *Hspa1b* by Primer v3.0 [44] for RT-qPCR amplification. The following sequences were constructed, a forward primer 5′-TGACGACCAAGATGAAGGAG-3′ and a reverse primer 5′-GTGATCTTGTTGGCCTTGCC-3′. Primers of *Atp5f1* and *Inmt* genes were adopted from previous studies [45,46].

Western blot method was performed to detect the relative protein expression levels of Hspa1b, Atp5f1, and Inmt. Briefly, the cells were lysed on ice with RIPA Lysis Buffer (Beyotime, Nanjing, China) and supplemented with 1% proteinase inhibitor (PMSF; Beyotime). Protein concentrations were determined using a BCA Protein Assay Kit (Thermo Fisher Scientific Co. LTD, USA). Samples containing 10 μg protein were separated on 10% sodium dodecyl sulfate-polyacrylamide gels (SDS-PAGE), and then electrotransferred onto a nitrocellulose membrane for 1 h using Bio-Rad Trans-Blot. The membrane was blocked with 5% non-fat milk in Tris-buffered saline (20 mM Tris-HCl, pH 7.6, 137 mM NaCl) containing 0.1% Tween-20 (TBST) for 30 min at room temperature and incubated at 4 °C overnight with the following primary antibodies: anti-rat Hspa1b, Atp5f1, or Inmt monoclonal antibody (10995-1-AP, 15999-1-AP, and ab181854, Proteintech, USA). The membranes were washed three times with TBS+ Tween 20 (TBST) for 10 min and incubated with a secondary horseradish peroxidase-conjugated goat anti-mouse IgG (H+L) antibody (Beyotime Institute of Biotechnology, Nanjing, China) for 1 h at room temperature. The antibody-antigen complexes were detected using Western blotting (WB) luminal reagent. The bands on the developed film were quantified with the Quantity One v4.6.2 software (Bio-Rad, Hercules, CA, USA). The GAPDH was used as a loading control for normalization.

### 2.11. Statistical Analysis

The student’s *t*-test was used for the analysis of changes in body temperature, dehydration rate, and the expression levels of genes and proteins in the BRL cell model between conditions. The Kruskal–Wallis test with Dunn’s Multiple Comparison Test was used for the analysis of changes in biochemical indicator levels among the control, H30, H60, and H120 groups. The statistical application used was SAS v9.4 (SAS Institute, Cary, NC, USA) and values with *p* < 0.05 were considered significant. Pearson correlation analysis was performed to investigate the relationship between the gene expression levels and five significantly different biochemical indicators levels.

## 3. Results

### 3.1. Thermal Behavior and Physiology Changes of HS-Rats

**Figure 2:**
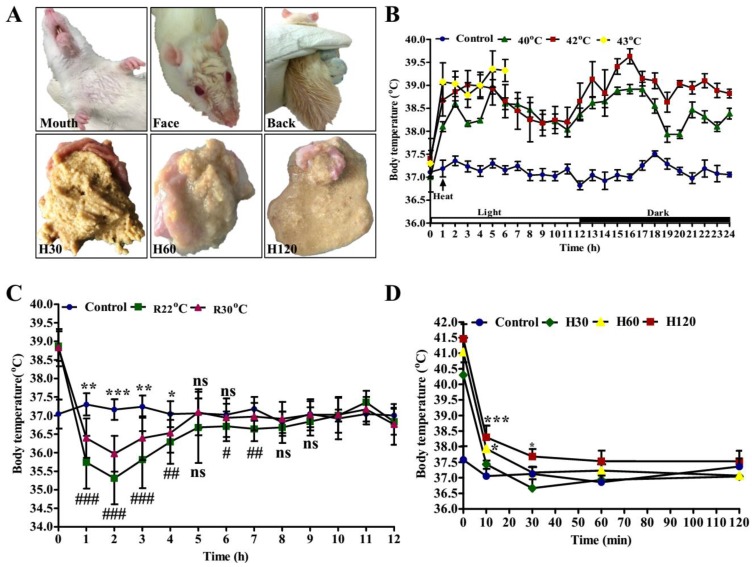
Behavior and physiological changes after heat stress (HS). (**A**) The behavior of rats at 42 °C HS. Figure 1A displays the sweating of rats and the state of their gastric content with prolonged HS time, sorted according to the order of sweating sites; (**B**) the body temperature of rats exposed to control (22 ± 1 °C) (*n* = 9), 40 °C (*n* = 9), and 42 °C (*n* = 18) conditions. The number 0 on x-axis means rats were at the control condition and 1-24 means 24 h, from 06:00 a.m. to 05:00 a.m. the next day; (**C**) body temperature recovery monitoring of the H120 group at ambient temperatures of 30 °C (R30 °C, *n* = 9) and control (R22 °C, *n* = 9). * and # represent the significant extent of R30 °C and R22 °C group temperature changes in comparison with the control, respectively. (**D**) The body temperature recovery of the rats in H30, H60 and H120 groups at 22 ± 1 °C, compared with control. *n* = 3 per group. Data is represented as mean ± standard deviation. The ns means *p* ≥ 0.05, *and # *p* < 0.05, ** and ## *p* < 0.01, *** and ### *p* < 0.001 (Student’s *t*-test).

The effects of HS on the behavior and body temperature in rats were presented in Figure 2. When the rats were placed under high ambient temperature, their activity was strengthened due to escaping behavior (hiding under the litter) and thermoregulatory grooming (saliva spreading and shortness of breath). With the prolonged HS time, the rats were sweating (upper panel of Figure 2A) and drinking more water, as observed by the state of gastric content (down panel of Figure 2A). We observed that the body temperature of rats increased with the rise of heat treatment intensity (Figure 2B). Compared with the controls (37.10 ± 0.17 °C), the average body temperatures of the rats housed under 40 °C (38.41 ± 0.31 °C), 42 °C (38.78 ± 0.37 °C) and 43 °C (38.97 ± 0.29 °C) within 24 h were significantly increased. Since three out of eight rats passed away at 43 °C heating for 6 h, their body temperature was not measured. The body temperature of the rats in control, 40 °C and 42 °C treatments at night were 0.071 °C, 0.11 °C, and 0.43 °C higher (*p* ≥ 0.05) than those during the daytime, respectively. 

During the recovery period, the body temperature recovery of HS-rats was comprised of initial hypothermia (30 °C, 35.97 ± 0.48 °C and 22 ± 1 °C, 35.31 ± 0.7 °C) and a consequent (~5 to 8 h) rise in body temperature (Figure 2C). It is worth noting that the hypothermia developed with the depth and duration of decreased ambient temperature was significantly enhanced in the 22 ± 1 °C as compared with the 30 °C recovery group. Rats in these two groups recovered to normal levels after 8 h and 5 h, respectively, by comparing body temperatures with the control group (*p* ≥ 0.05). 

On the basis of the above observations, 42 °C was chosen as the ambient temperature of HS, and the body temperature of treated rats was detected at H30, H60, and H120, respectively. Post heat treatment, the rats recovered at 22 ± 1 °C (Figure 2D), in which we observed rats in the H120 group suffered more severe HS. H120 was used as the experimental condition in this study.

### 3.2. Significant Biochemical Indicator Changes Induced by HS for Different Durations

**Figure 3:**
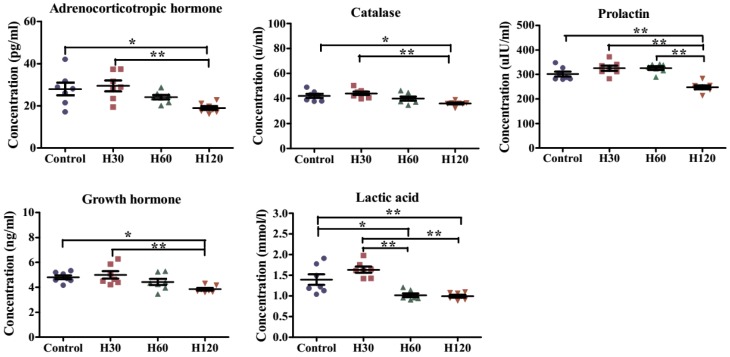
Effect of different heat stress (HS) durations on five kinds of biochemical indicators levels in rat blood samples. There are seven rats in each group. Data is presented as mean ± standard deviation. The * indicates a significant difference among treatment groups. * *p* < 0.05 and ** *p* < 0.01.

The changes of 16 biochemical indicators of blood were time dependent (Figure 3 and Figure S1). The concentrations of adrenocorticotropic hormone (27.96 ± 7.95 vs. 18.95 ± 2.42 pg/mL), catalase (42.00 ± 4.18 vs. 36.00 ± 2.04 µ/mL), prolactin (301.86 ± 26.48 vs. 248.16 ± 21.03 µL/mL), growth hormone (4.81 ± 0.42 vs. 3.85 ± 0.28 ng/mL), and lactic acid (1.39 ± 0.34 vs. 1.00 ± 0.09 mmol/L) in H120 groups were significantly lower than those in the control groups (Figure 3). Furthermore, the concentrations of adrenocorticotropic hormone, catalase, prolactin, growth hormone, lactic acid, C-reactive protein, and lipid peroxidase in H120 group were 10.51 pg/mL, 8.05 µ/mL, 77.36 µL/mL, 1.15 ng/mL, 0.64 mmol/L, 3.07 nmol/mL, and 1.04 ng/mL lower (*p* < 0.01) than those in H30 group, respectively (Figure 3 and Figure S1). Additionally, the prolactin content of the H120 group was significantly lower than that of the H60 group (Figure 3). The changes in other biochemical indicators under different heat treatment conditions are shown in Figure S1. Therefore, more severe HS responses occurred in H120 rats. On the basis of the rat body temperature and biochemical indicators in blood, H120 was used as the HS condition in the rat model. 

### 3.3. Dehydration Rate Changes in Rats before and after H120

For further physiological changes associated with HS response, the dehydration rate of rats in the H120 group was calculated via the change in body weight before and after H120 by electronic scale (Figure S2). The body weight of the rats after H120 significantly decreased by 13.4 g, relative to the body weight before HS, which was consistent with the previous study [34]. 

The water loss of the rats was calculated as follows:$dehydration\;rate=\frac{H120\;weight-Control\;weight}{Control\;weight}\times 100\%$. In the current study, the dehydration rate of rats was 6.58%, which indicated the rats were thermally regulated by evaporative heat [47].

### 3.4. The Whole Genome-Wide Transcriptional Changes of HS-Treated Rats

The average of the total paired-end reads of the samples was 31,227,548 bp (ranging from 25,853,489 to 48,529,502 bp), and 31,759,037 bp after data filtering with a GC content of 50.81% (Table S2). The average percentages of Q20 and Q30 were 95.06% and 89.10%, respectively. An average of 9.46 G bases was successfully mapped to the newest version of the rat reference genome (Rattus norvegicus 6.0). Bioinformatic analysis for high throughput transcriptome data revealed a total of 28,283 genes commonly annotated in blood, liver, and adrenal gland tissues. On the basis of the FPKM values, the correlations of four and five repeats for blood, liver, and adrenal gland tissues under H120 were measured (Figure S3). The results showed there were good correlations among these repeats. 

**Figure 4:**
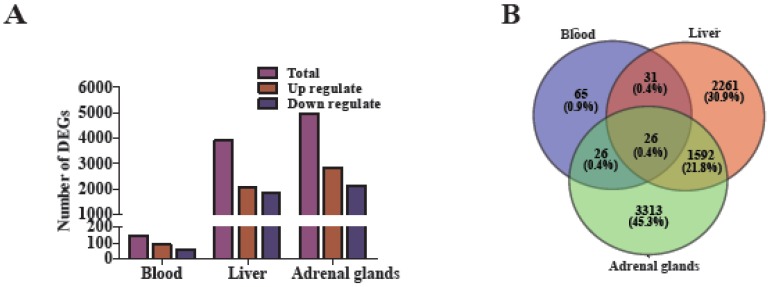

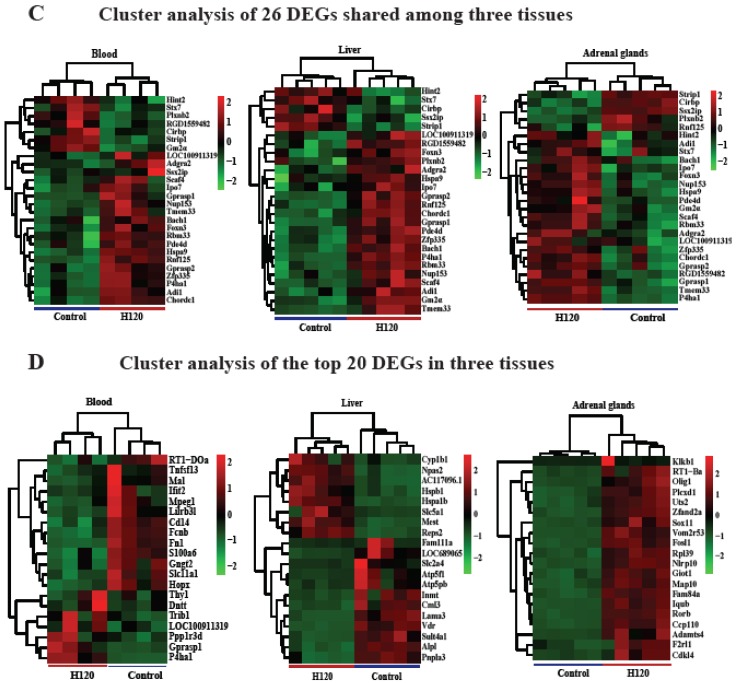
Comparisons and Pheatmap of the shared and top 20 DEGs in blood, liver, and adrenal gland tissues. (**A**) Total number of DEGs (*q* < 0.05; purple), upregulated (red) and downregulated (blue) in blood (*n* = 4), liver (*n* = 5), and adrenal glands (*n* = 5) when rats underwent H120; (**B**) the Venn diagram displays the common and tissue specific DEGs among blood, liver, and adrenal glands; (**C**) hierarchical clustering analysis of the commonly identified 26 DEGs from blood, liver and adrenal gland tissues; (**D**) hierarchical clustering analysis of the top 20 DEGs from blood, liver, and adrenal gland tissues. Upregulated and downregulated refers to the expression levels of genes in H120 rats were higher and lower than the controls, respectively. The log _(10+1)_-transformed FPKM values (rows) are clustered using hierarchical clustering, and the samples are grouped according to the similarity of gene expression profiles.

A total of 149, 3909, and 4953 DEGs were identified in the tissues of blood, liver, and adrenal glands in the comparison of H120 vs. Controls, respectively. The DEGs identified in each tissue according to various criteria of *Q* value and fold change values is described in Table 1. A total of 146 DEGs were known and three DEGs were novel in the blood samples (Table S3), among which 90 DEGs were upregulated (Figure 4A), while 56 DEGs were downregulated. In the liver, 3909 DEGs containing seven novel DEGs were identified (Table S4), among which 2037 genes were upregulated and 1872 genes were downregulated. Adrenal gland tissues had the highest number of DEGs (Table S5), with eight novel genes. The RNA-seq data showed more DEGs in the adrenal glands, followed by liver and blood, therefore the adrenal glands respond more strongly to HS (Figure 4A). 

### 3.5. The Key DEGs in Different Rat Tissues in Response to HS 

We observed 26 common DEGs among the three tissues using the Venn diagram (Figure 4B), out of which 16 were upregulated, and two were downregulated, while the remaining eight genes showed various patterns (Table S6). Furthermore, 57 DEGs were shared between blood and liver tissues, 52 DEGs were distributed between blood and adrenal glands, and 1644 DEGs between liver and adrenal gland tissues. Whilst, 65, 2261, and 3313 DEGs were identified as tissue-specific in blood, liver, and adrenal gland tissues, respectively. Out of them, 38, 1169, and 1918 DEGs were upregulated, and 27, 1092, and 1395 DEGs were downregulated, respectively (Figure 4B).

Cluster analysis of 26 DEGs shared among the three tissues are shown in Figure 4C (left, middle, and right panels are blood, liver, adrenal glands, respectively) and their expression levels are displayed in Table S6. Among these DEGs, *P4ha1* gene was expressed at very high levels (ranging from 10.46- to 12.26-fold in the comparison of H120 vs. Control). Eight DEGs, including *Bach1*, *Plxnb2*, *Stx7*, *Hspa9*, *Chordc1*, *Pde4d,* and *Gm2α* regulate the response to stress, especially the *Chordc1* gene, which was involved in HS response. Nine genes (*Nup153*, *Plxnb2*, *Stx7*, *Hspa9*, *Chordc1*, *Pde4d*, *Gm2α,* and *Rnf125*) are involved in activating the innate and adaptive immune response, and therefore can defend tissues against HS by activating the immune system. The remaining DEGs were involved in the regulation of cell morphogenesis (*Strip1*), endoplasmic reticulum stress (*Tmem33*), hepatic glucose utilization (*Foxn3*), and the establishment of the blood–brain barrier (*Adgra2*). 

Furthermore, the expression profiles of the top 20 DEGs within each tissue were well illustrated, as shown in Figure 4D. When rats were exposed to H120, more than half of the top 20 DEGs were downregulated in blood and liver; however, all of the DEGs were upregulated in the adrenal glands. The largest increases in the expression levels of *P4ha1*, *Hspa1b,* and *Vom2r53* were found in blood, liver, and adrenal gland tissues, respectively, and the fold change values of the top 20 DEGs in the adrenal glands were far greater than those in blood and liver (Table S7). 

### 3.6. Functional Annotation of DEGs Involved in HS

**Figure 5:**
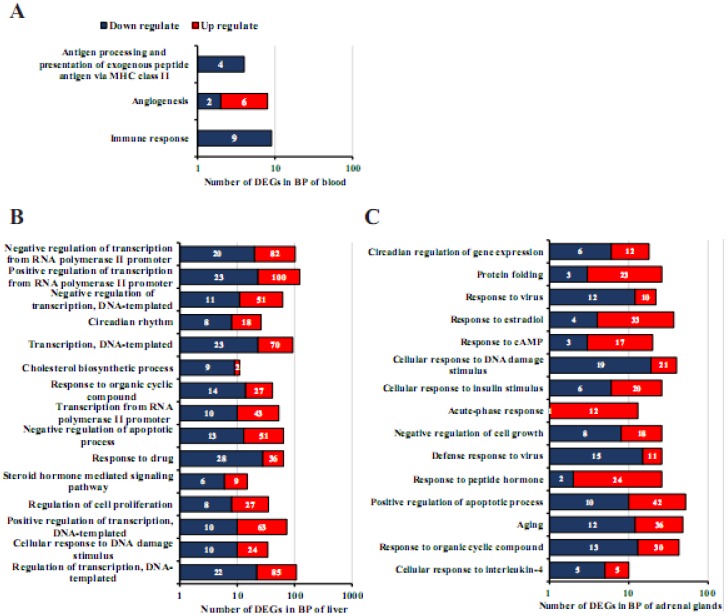
Significantly enriched biological process (BP) terms of blood, liver, and adrenal gland tissues in H120 vs. Control groups. (**A**, **B** and **C**) The top 15 significantly enriched BP terms identified in blood, liver, and adrenal gland tissues. The number of upregulated (red) and downregulated (blue) DEGs (*q* < 0.05 and absolute log_2_ (fold-change) > 1) enriched BPs in blood, liver, and adrenal glands when H120 was compared to control groups. The x-axis coordinates are represented on a logarithmic scale with a radius of 10. The BP terms (up to down) were sorted by *q* values from small to large.

The GO analysis of the 146 DEGs (*q* < 0.05) in blood, 1550, and 2023 DEGs in the liver and adrenal glands, respectively, (*q* < 0.05 and |fold-change| > 2) were carried out for the functional classification of HS-response genes. The GO analysis is summarized in Figure 5 and Table S8, which classifies DEGs into the following three major categories: biological process (BP), cellular component (CC), and molecular function (MF). The 3, 121 and 79 BPs were significantly enriched in blood, liver, and adrenal glands, and no BPs were shared among blood and other tissues (liver and adrenal glands) (Table S8). In blood, all the DEGs enriched in antigen processing and presentation of exogenous peptide antigen via MHC class II (GO: 0019886) and immune response (GO: 0006955) were downregulated (Figure 5A and Table S8). The CC category revealed the DEGs of blood, liver, and adrenal glands were only commonly grouped under cytoplasm (GO: 0005737) and membrane (GO: 0016020) (Figure S4), and six CC terms including the extracellular exosome (GO: 0070062) were shared between blood and adrenal glands (Figure S4A,C and Table S8). Moreover, 36 DEGs (14 upregulated and 22 downregulated) in blood and 314 DEGs (234 upregulate and 80 downregulated) in the adrenal glands were identified in the composition of extracellular exosome CC term. Among these genes, *Pteges3*, *Strip1*, *Tmem33,* and *Cct6A* were shared between blood and adrenal glands. The previous study found that HS triggered an increase of *Pteges3*, a decline of gamete/embryo transport through the oviduct and, consequently, weakening the reproductive function of cows [48]. To the best of our knowledge, no study has reported the association of *Tmem33, Cct6A,* and *Strip1* with HS. The top three DEGs (*S100a6*, *Cd14,* and *Fn1*) in blood were all downregulated, while the top three DEGs (*Klkb1*, *Hspb1,* and *Hsph1*) in adrenal glands with fold change ranges from 21.68 to 24.46 were upregulated.

The KEGG pathway analysis revealed 8, 38, and 25 pathways significantly enriched in blood, liver, and adrenal gland tissues, respectively, with no shared pathways (Table S9). In blood, the hematopoietic cell lineage pathway (rno04640) contained six DEGs and was ranked as the first pathway followed by the osteoclast differentiation pathway (rno04380) with seven DEGs. Moreover, five pathways including, cancer (rno05200), amoebiasis (rno05146), antigen processing and presentation (rno04612), chemokine signaling (rno04062), and leukocyte transendothelial migration (rno04670) pathways, were interconnected and directly involved in the inflammatory host defense, which suggests that multiple innate immunity and inflammation-related pathways could be involved in the response to HS. The 15, 135, and 293 DEGs (*q* < 0.05 and |fold-change| > 5) of blood, liver, and adrenal glands were also included in the GO and KEGG analysis (Figure S5). 

### 3.7. The GGI and PPI Networks of DEGs Enriched in Extracellular Exosome Post HS

**Figure 6:**
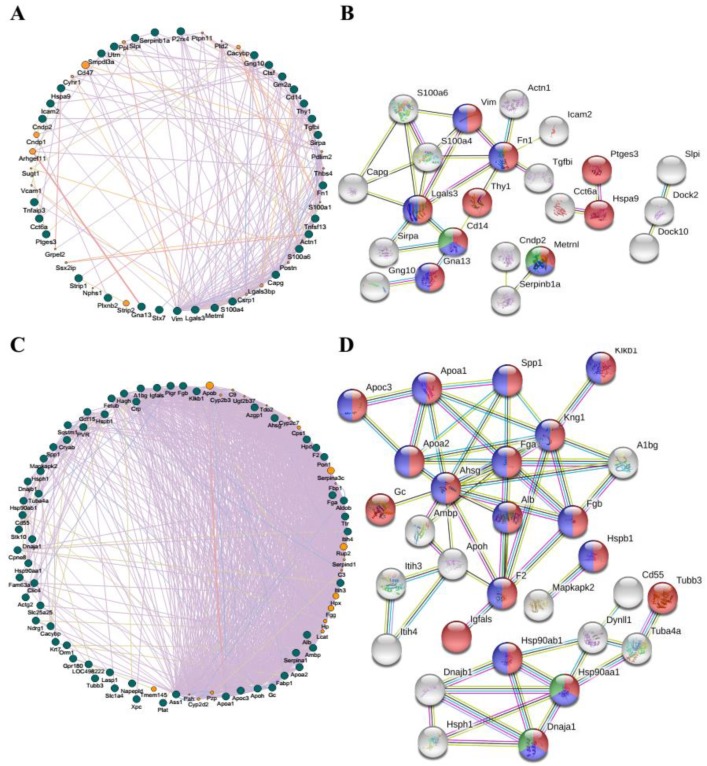
Gene–gene interaction (GGI) and protein–protein interaction (PPI) predicted networks of DEGs enriched in the extracellular exosome post heat stress. (**A**) The GGI network analysis was performed based on the BPs of all 36 genes in blood produced by GeneMANIA; (**B**) The PPI network analysis of 23 genes in blood produced by STRING.11 (confidence 0.4). The proteins of the remaining 13 genes were disconnected; (**C**) The GGI network performed based on the BPs of all 69 genes in the adrenal glands produced by GeneMANIA; (**D**) The PPI network of 29 genes in the adrenal glands produced by STRING.11 (confidence 0.9). The proteins of the remaining 40 genes were disconnected. The GGI network was sorted by degree. A total of 314 genes in the adrenal glands were enriched in the extracellular exosome term, of which 69 genes with log_2_ (fold-change) >2 were subjected to PPI network analysis. In (A) and (C), the dark green nodes represent the query genes and the orange nodes represent the gene’s function has been validated. Different colored lines represent four types of evidence used in predicting associations: red, co-expression; blue, co-localization; green, physical interactions; grey, pathway; and yellow, shared protein domains. In (B) and (D), each node represents one protein. All the annotated proteins are shown as white nodes and colored nodes represent the proteins involved in stress-related BPs: red, response to a stimulus; blue, response to stress; and green, response to heat (B) or response to temperature stress (D). Different colored lines represent four types of evidence used in predicting associations: yellow, text mining evidence; purple, experimental evidence; light blue, database evidence; and black line, co-expression evidence.

Heat stress normally induces genome-wide variations in genes and proteins; thus, the GGI and PPI analyses of 36 and 314 DEGs in blood and adrenal glands enriched in the extracellular exosome pathway were performed, respectively (Figure 6). The GGI network for all 36 genes in blood illustrated they were highly interconnected (Figure 6A). A subset of 23 proteins was moderately interconnected (Figure 6B). The proteins encoded by *Vim*, *Lgals3*, *Fn1*, *Thy1*, *Cd14*, *Gnal13*, *Ptges3*, *Hspa9,* and *Metrnl* are involved in the response to stimulus process, of which, the Vim, Lgals3, Fn1, Cd14, Gnal13, and Metrnl proteins regulate stress responses. Furthermore, the Cd14 and Metrnl proteins have been reported to play an important role in the HS response [49,50]. Only 69 out of 314 genes in the adrenal glands (*q* < 0.05 and |fold-change| > 4) were used for GGI analysis. Results demonstrated that these genes and 29 proteins identified in the STRING database were highly interconnected (confidence 0.9, Figure 6C,D). Moreover, 18 proteins were enriched in the response to stimulus BP, 15 of which were involved in stress response, and two, including Hsp90aa1 and Dnaja1, were engaged in the regulation of HS (Figure 6D).

### 3.8. Regression Analysis of the DEG Levels with the Biochemical Indicator Levels in Blood

**Figure 7:**
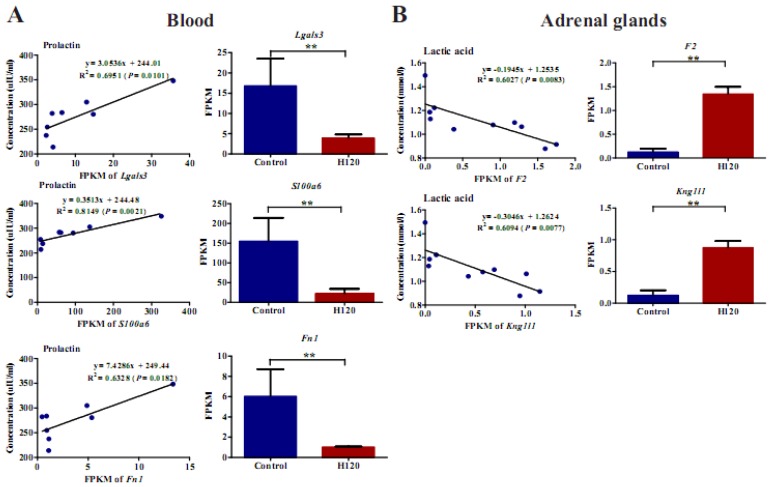
Regression analysis of the significantly DEGs (x-axis) with the biochemical indicator levels of blood (y-axis). (**A**) The regression analysis of the expression levels of *S100a6*, *Fn1,* and *Lgals3* in blood with the biochemical indicator levels (*n* = 4 per group); (**B**) the regression analysis of the expression levels of *F2* and *Kng1l1* in the adrenal glands with the biochemical indicator levels (*n* = 5 per group). *p* < 0.05 means significant regression. ** represents *p* < 0.01.

In order to explore the vital role of DEGs that comprise the extracellular exosome in HS response, the top 15 DEGs and their encoded proteins (linked protein number > 4) were selected for regression analysis with biochemical indicators in blood (Figure 7 and Figure S6). The expression levels of the DEGs identified in blood were positively correlated with changes in biochemical indicators, while the opposite trend was found in the adrenal glands. Genes identified in blood including, *Lgals3*, *S100a6,* and *Fn1* had a significant (R^2^ *>* 0.6) positive regression relationship with prolactin, while *F2* and *Kngll1* in the adrenal glands had a significant (R^2^ *>* 0.6) negative regression relationship with lactic acid (Figure 7). Additionally, *vim*, *S100a4,* and *Cd14* in blood and *Ahsg*, *F2*, *Albg*, *Fga*, *Fgb*, *Apoa1,* and *Apoh* in the adrenal glands were strongly correlated (R^2^ > 0.5) with various biochemical indicators, such as prolactin, lactic acid, adrenocorticotropic hormone, and growth hormone (Figure S6). 

### 3.9. Validation of RNA-Seq Data by qRT-PCR

**Figure 8:**
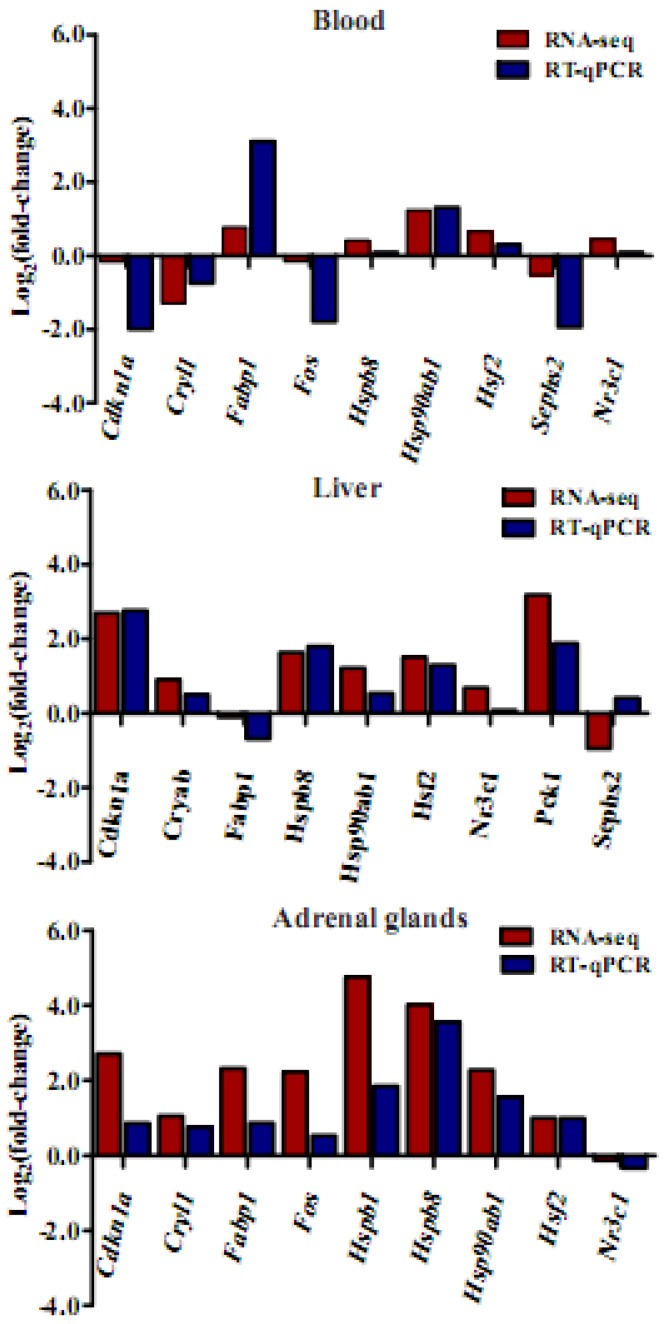
Gene expression patterns between RT-qPCR and RNA-seq data in blood, liver, and adrenal glands.

In order to verify the accuracy of RNA-seq data, nine genes were selected for RT-qPCR validation in blood, liver, and adrenal glands (Figure 8). Our results showed the expression patterns of these genes generated from RNA-seq were consistent with the results of RT-qPCR. The Pearson correlation of the genes in blood, liver, and adrenal glands between RNA-seq analysis and RT-qPCR were 0.89, 0.91, and 0.89 (*p* < 0.01), respectively.

### 3.10. Confirming the Variation of Hspa1b, Atp5f1, and Inmt, as well as their Proteins Induced by HS in Vitro 

**Figure 9:**
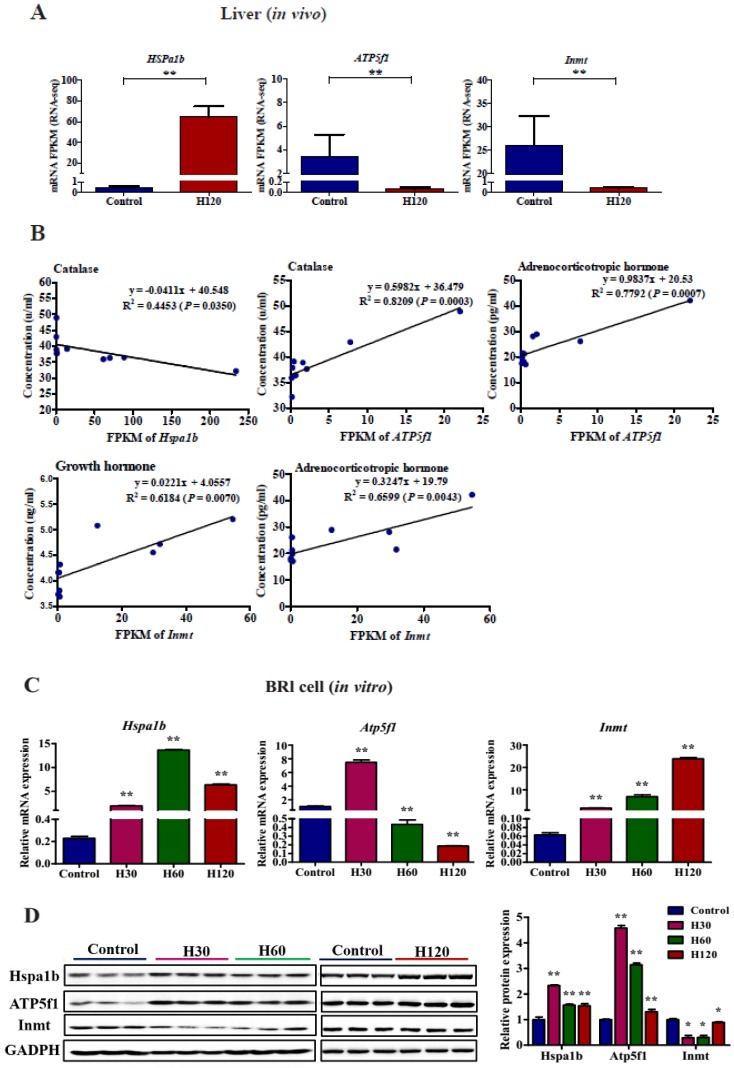
Effect of heat stress (HS) on the mRNA and protein expression of *Hspa1b*, *ATP5f1* and *Inmt* in rats. (**A**) The mRNA expression levels of *Hspa1b*, *ATP5f1,* and *Inmt* in liver (*in vivo*). The expression levels of *Hspa1b*, *ATP5f1,* and *Inmt* in the liver tissue of H120-treated rats and the time-matched control group (*n* = 5); (**B**) regression analysis of the top 3 significant DEG expression levels (x-axis) in the liver and biochemical indicator levels of significant changes in serum (y-axis); (**C**) the expression level of *Hspa1b*, *ATP5f1,* and *Inmt* in BRL cell (*in vitro*). The control BRL cells were cultured at 37 °C and the HS-cells were treated under H30, H60, and H120 conditions. Three independent experiments were performed for each treatment; (**D**) the effect of HS on the expression of Hspa1b, ATP5f1, and Inmt proteins in BRL cells by WB and their expression relative to GAPDH. All data are presented as mean ± SEM. Each bar indicates the standard error of means. *p* < 0.05 is a significant regression. * represents *p* < 0.05 and ** represents *p* < 0.01.

Considering the expression levels of *Hspa1b*, *Atp5f1,* and *Inmt* in liver tissue and their significant regression relationship with the biochemical indicators in blood, the expression levels of these genes and proteins in HS BRL cells *in vitro* were further detected using RT-qPCR and WB techniques (Figure 9). A significant negative regression relationship was found between *Hspa1b* and catalase (Figure 9B). Significant positive regressions were observed between *Atp5f1* and two biochemical indicators (catalase and adrenocorticotropic hormone). In addition, a positive regression between *Inmt* and two biochemical indicators (growth hormone and adrenocorticotropic hormone) was noticed (Figure 9B). 

The expression patterns of *Hspa1b*, *Atp5f1,* and *Inmt* in BRL cells were different under various durations of HS (Figure 9C). The expression levels of *Hspa1b* in HS groups were all higher (*P* < 0.01) then in the control group. As the duration of HS increased the expression level of *Hspa1b* increased from H30 to H60, and then decreased slightly at H120. Compared to the control, the expression level of *Atp5f1* significantly increased to 7.4 in the H30 group, but significantly decreased to 0.43 and 0.19 in the H60 and H120 groups, respectively. The expression level of *Inmt* continued to increase significantly from 22 ± 1 °C to H120. The data shown in Figure 9A,C indicate that the *in Vivo* and *in vitro* regulation of *Hspa1b* and *Atp5f1* were stable throughout HS. However, *Inmt* showed an opposite trend *in vivo* and *in vitro*, which warrants further research by increasing samples size and technique repeats. The expression of Hspa1b first increased and then decreased slightly at H60 and H120, which is consistent with other HSP family expression levels under HS [51] (Figure 9D). The relative protein level of Atp5f1 in HS groups was all higher (*P* < 0.01) than that in control groups (Figure 9D) and combined with the expression level of *Atp5f1* gene at H60 and H120 conditions (Figure 9C), demonstrating that HS has a delayed effect on the expression of proteins. It is interesting to note, the changes in the Inmt protein under various HS durations (declined at H30 and H60, and then increased marginally at H120) indicate a significantly lower (*P* = 0.02) level of Inmt in HS groups as compared with the control. 

## 4. Discussion

A further understanding of how animals cope with HS from a physiological and biochemical level through studying the effect on different body tissues is essential for understanding thermo-tolerance and the discovery and validation of potential biomarkers. In the present study, using a well-established HS-rat model, we found that mild HS can lead to physiological, biochemical, and transcriptomic changes in blood, liver, and adrenal gland tissues. To the best of our knowledge, the current study is the first to investigate the altered set of expression levels in genes coding for extracellular exosome composition-related proteins in blood and adrenal gland tissues under HS treatment. These findings provide insight into exosome secretion that can occur following short and mild heat shock. The genes identified could be important candidate markers of HS in livestock. 

Our first main finding is that the extracellular exosome present in blood and adrenal glands can be an important regulator of HS. Previous studies assessed the degree of HS in livestock based on physiological (e.g., body temperature [52], salivary or respiratory rate [53], and biochemical indicators (e.g., cortisol, adrenocorticotropic hormone, prolactin) [10,54]). However, the genetic background, HS intensity and duration can affect the stability and reliability of the response to HS. In the current study, we use changes in body temperature and biochemical indicators as measures for a HS rat model. Furthermore, we explored the relationship between DEGs coding for extracellular exosomal proteins and blood biochemical indicators, providing a more reliable HS assessment. Exosomes serve as invaluable regulators of the immune response [55] and regulate the acute stress response by modulating innate immunity [56]. HS can modify the composition and function of exosomes [57], suggesting that exosomes could be potential markers for the assessment of HS intensity. Exosomes have been discovered in blood, urine, and breast milk, etc. [58,59,60], however, to date exosomes have not been identified in adrenal glands. The secretion of exosomes can be detected via the presence of several reliable markers, including transmembrane domain proteins of CD9, CD63, CD81, and CD82 [61,62]. In the current study, we identified *Cd63* and *Cd82* (*q* ≥ 0.05) in blood, and *Cd63* and *Cd81* (*q* < 0.01, 1.3 < Log_2_ (fold change) < 2) in the adrenal glands, which indirectly demonstrates the presence of exosomes in blood and adrenal glands of rats under HS. Notably, our study is the first to discover DEGs involved in exosome composition in the adrenal glands. 

**Figure 10:**
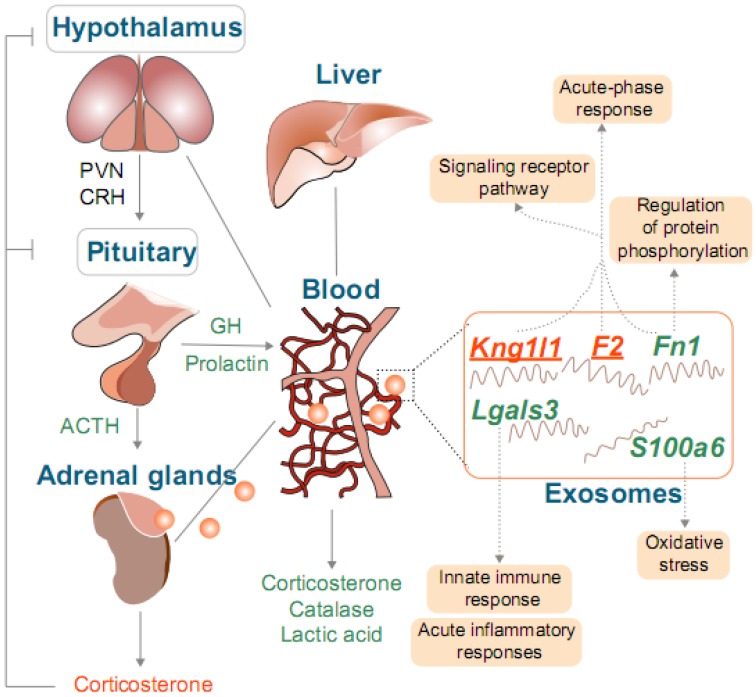
Model of the influence of heat stress (HS) on Sprague Dawley (SD) rat. The left panel of this figure shows the basic regulation mechanism of the hypothalamus-pituitary-adrenal gland axis (HPA axis). PVN is the paraventricular nucleus and CRH is the corticotropin-releasing hormone. The blood, liver, and adrenal gland tissues (no grey box) were the target tissues in the present study. The adrenocorticotropic hormone, growth hormone, prolactin, catalase, and lactic acid were significantly different in the blood of H120 rats as compared with the control. Furthermore, the biochemical indicators in orange and green represent an increase and decrease in the concentration of the H120 as compared with the control groups. The corticosterone changes were detected in our previous study and compared with the control group. The concentration of corticosterone increased at H120 in adrenal glands (orange mark) but decreased in blood (green mark). The solid orange balls represent the extracellular exosomes. The genes listed are the candidate genes selected in blood and adrenal glands (underlined). Genes in orange and green represent upregulated and downregulated, respectively. These genes are linked with their respective pathways (light orange box).

Genes identified in blood including, *Lgals3*, *S100a6,* and *Fn1*, and in the adrenal glands, *F2* and *Kng1l1,* were identified as candidate genes in the composition of exosomes for HS response. These genes play a central role in immunity and oxidative stress as summarized in Figure 10.

We noticed that *Lgals3* in blood decreased about 76.9% in H120 rats (Figure 7A), which is consistent with previous findings identifying approximately 50% of *Lgals3* downregulated when exposed to HS [63]. It has been demonstrated that the expression of Lgals3 (galectins) in pituitary adenomas is positively associated with the prolactin level in pituitary tumorigenesis [64]. Our results indicate a positive correlation between *Lgals3* and prolactin, however, the regulatory function of *Lgals3* on prolactin under HS has not been illustrated. The S100a6 competes with Hsp90 for binding to sgt1 [65] and it influences the translocation of Sgt1 in human epidermoid carcinoma cells during HS [66]. It has also been reported that an increase of S100a6 is normally present in some neurodegenerative diseases (e.g., Alzheimer’s disease) depending on the regulation of the HPA-axis. However, the interaction of S100a6 with hormones is unclear. In our study, the expression of *S100a6* in blood declined and was positively correlated with prolactin levels (Figure 7A). Therefore, our study provides a baseline for revealing the role of *S100a6* in hormonal regulation throughout HS. Fibronectin (*Fn1*) is a stress-responsive and functional *Hsf1* target gene, whose promotor has three putative heat shock elements that can regulate the expression of *Hsf1* [67]. Furthermore, prolactin can stimulate the adhesion of peripheral blood mononuclear cells to immobilize intercellular adhesion molecule-1 and fibronectin [68]. Therefore, our study identifies the relationship between *Fn1* and prolactin in blood during HS, indicating HS can influence the adhesion and migration of cells. In summary, our study is the first to discover and emphasize the importance of exosomes during the response to HS and proposed several candidate genetic markers. However, the results from previous studies and the present study are descriptive and do not identify the functional role that genes play in HS. Therefore, the detection of indicators in exosomes and the validation of exosome function during HS is warranted.

A second highlight of this study is that the transcript profiles of blood, liver, and, adrenal glands demonstrate *P4ha1* could be the response of a dominant gene to HS. Notably, *P4hα1* had the largest fold change among all 26 DEGs, with an average fold change of 11.44 in the three tissues (Figure 4C and Table S6); furthermore, it was DEG of the highest fold change in blood (Figure 4D). Heat stress can induce endoplasmic reticulum (ER) stress in various mammalian cells through disturbing the homeostasis and, consequently, accumulating unfolded or misfolded proteins [69]. It has been shown that *P4hα1* was upregulated during ER stress [70], indicating that HS triggered ER stress in our research. In addition, the functional annotation analysis demonstrated that the *P4hα1* was significantly enriched in the oxidation-reduction process (GO: 0055114) and the metabolism pathway during HS (Table S8). Therefore, *P4hα1* could be a candidate genetic marker for HS research and highly recommended for future research. Blood is a typical sample in clinical and experimental research and compared to other models such as liver and adrenal glands, it is noninvasive, easy to collect, and has the ability to provide short-term measures of stress. This will be beneficial to the future application of genetic markers in the breeding of heat-resistance varieties. We established a HS rat model according to the changing patterns of the biochemical indicators in blood and confirmed the key markers from the transcriptomic profiles among blood, liver, and adrenal glands. Therefore, our study will provide noninvasive and more reliable HS markers for heat-resistant breeding programs.

## 5. Conclusions

In summary, the physiological and biochemical phenotypes, and the global transcriptomic profiles across different tissues in rat models under short-term, mild HS revealed the spatiotemporal variability and complexity of HS response. The transcriptome profile of adrenal glands under HS was first revealed for the verification of the HPA axis effect on the regulatory function of an organism. Our study highlighted the importance of exosomes in the regulation of HS, which will be a benefit for understanding the mechanisms involved in exosomal modifications and provided valuable insight into the possibility of using exosomes as HS markers and prevention tools. This study provided important insight into future efforts attempting to improve species abilities to withstand HS through genome-wide association studies and breeding.

## Figures and Tables

**Table 1:** Numbers of differentially expressed genes (DEGs) identified in blood, liver, and adrenal glands post H120 treatment.

Criteria	Expression Models	H120 vs. Control		
		Blood	Liver	Adrenal Glands
*q* < 0.05	Total	149	3909	4953
	Up	90	2037	2821
	Down	59	1872	2132
*q* < 0.05,|Fold change| > 2	Total	146	1550	2023
	Up	90	985	1204
	Down	56	565	819
*q* < 0.05,| Fold change | > 5	Total	15	135	293
	Up	4	85	256
	Down	11	50	37
*q* < 0.05,| Fold change | > 7	Total	2	65	173
	Up	1	36	161
	Down	1	29	12
*q* < 0.05,| Fold change | > 10	Total	no	33	89
	Up		17	85
	Down		16	4

## Supplementary Materials

The following are available online at https://www.mdpi.com/2073-4425/11/3/306/s1, Figure S1: related to Figure 3. Effect of different heat stress (HS) durations on 11 kinds of biochemical indicators levels in blood of rats. Figure S2: The dehydration rate changes of the rats before and after 42 °C heat stress for 120 min (H120). Figure S3: The Pearson correlation coefficient (PCC) analysis of samples in blood (A), liver (B) and adrenal gland (C) tissues. Figure S4: Significantly enriched cellular component (CC) and molecular function (MF) terms of blood, liver and adrenal glands in H120 vs. Control comparisons. Figure S5: Significantly enriched biological process (BP) terms of DEGs with *q* < 0.05 and absolute log2(fold-change) > 2.32 in liver and adrenal glands in H120 vs. Control comparisons. Figure S6: Regression analysis of the significantly differentially expressed gene (DEGs) levels (x-axis) with the biochemical indicators levels of serum (y-axis). Table S1: The primers used for qRT-PCR in blood, liver and adrenal gland tissues. Table S2: The statistics summary of reads generated from 28 RNA-seq libraries in control and H120 groups. Table S3: The differentially expressed genes (DEGs) detected in blood when H120 vs. Control. Table S4: The differentially expressed genes (DEGs) detected in liver when H120 vs. Control. Table S5: The differentially expressed genes (DEGs) detected in adrenal glands when H120 vs. Control. Table S6: The statistical summary of 26 shared differentially expressed genes (DEGs) identified in blood, liver and adrenal gland tissues. Table S7: Review of the top 20 differentially expressed genes (DEGs) in blood, liver and adrenal glands of heat stressed-rats relative to the control non-heat stressed group using RNA-seq (ranked according to fold change [smallest to largest] within each tissue, the top 1 DEGs in each tissues were marked in yellow). Table S8: Significantly enriched gene ontology (GO) terms from gene set analyses in blood, liver and adrenal gland tissues at H120. Table S9: The summary of significantly enriched Kyoto Encyclopedia of Genes and Genomes (KEGG) pathways in blood, liver and adrenal gland tissues at H120.
