# Supplementary material for: Heat Stress Impairs the Physiological Responses and Regulates Genes Coding for Extracellular Exosomal Proteins in Rat
Source: Genes (Basel). 2020 Mar 13;11(3):306. doi: 10.3390/genes11030306 (PMC7140893; doi:10.3390/genes11030306)
Supplement: Supplementary file 1 [file genes-11-00306-s001.zip › genes-717632-supplementary/Supplementary_files.docx]

**Heat stress impairs the physiological responses and regulates genes coding for extracellular exosomal proteins in rat**

**Jinhuan Dou ^1^, Adnan Khan ^1^, Muhammad Zahoor Khan^1^, Siyuan Mi ^1^, Yajing Wang ^2^, Ying Yu ^1^, Yachun Wang ^1,*^**

1. Key Laboratory of Animal Genetics, Breeding and Reproduction, MARA, National Engineering Laboratory for Animal Breeding, College of Animal Science and Technology, China Agricultural University, 100193, Beijing, P.R. China; doujinhuan_cau@163.com (J.D), [dr.adnan93@cau.edu.cn](mailto:dr.adnan93@cau.edu.cn) (A.K), [zahoorkhattak91@163.com](mailto:zahoorkhattak91@163.com) (M.Z.K), [caumsy@163.com](mailto:caumsy@163.com) (S.M), [yuying@cau.edu.cn](mailto:yuying@cau.edu.cn) (Y.Y) and [wangyachun@cau.edu.cn](mailto:wangyachun@cau.edu.cn) (Y.W)
2. State Key Laboratory of Animal Nutrition, Beijing Engineering Technology Research Center of Raw Milk Quality and Safety Control, College of Animal Science and Technology, China Agricultural University, 100193, Beijing, P.R. China, wangyajing_cau@163.com (Y.W),

***** Correspondence: [wangyachun@cau.edu.cn](mailto:wangyachun@cau.edu.cn) (Y.W)

**Table legends.**

**Table S1. The primers used for qRT-PCR in blood, liver and adrenal gland tissues.**

**Table S2. The statistics summary of reads generated from 28 RNA-seq libraries in control and H120 groups.**

**Table S3. The differentially expressed genes (DEGs ) detected in blood when H120 vs. Control.**

**Table S4. The differentially expressed genes (DEGs) detected in liver when H120 vs. Control.**

**Table S5. The differentially expressed genes (DEGs) detected in adrenal glands when H120 vs. Control.**

**Table S6. The statistical summary of 26 shared differentially expressed genes (DEGs) identified in blood, liver and adrenal gland tissues.**

**Table S7. Review of the top 20 differentially expressed genes (DEGs) in blood, liver and adrenal glands of heat stressed-rats relative to the control non-heat stressed group using RNA-seq (ranked according to fold change [smallest to largest] within each tissue, the top 1 DEGs in each tissues were marked in yellow).**

**Table S8. Significantly enriched gene ontology (GO) terms from gene set analyses in blood, liver and adrenal gland tissues at H120. White-colored cells represent the GO terms common to all the three organs (consensus heat-stress response [HSR]); gray-colored cells contain terms common to two organs (common HSR); and the remaining cells are color-coded by organ (red = blood only; orange = liver only; blue = adrenal glands only).**

**Table S9. The summary of significantly enriched Kyoto Encyclopedia of Genes and Genomes (KEGG) pathways in blood, liver and adrenal gland tissues at H120. Gray-colored cells contain terms common to two organs (common HSR); and the remaining cells are color-coded by organ (red = blood only; orange = liver only; blue = adrenal glands only).**

**Figure legends**

**Figure S1, related to Figure 3. Effect of different heat stress (HS) durations on 11 kinds of biochemical indicators levels in blood of rats.** Control was kept at room temperature (22 ± 1 ^o^C, relative humidity [RH] (%): 50 %); heat stressed were acclimated to 42 ^o^C and RH 50% for 30 min (H30), 60 min (H60) and 120 min (H120), seven rats in each group. Data is presented as mean ± standard deviation. The * indicates significant difference among treatment groups. * *P* < 0.05, ** *P* < 0.01.

**Figure S2. The dehydration rate changes of the rats before and after 42 ^o^C heat stress for 120 min (H120).** Data is presented as mean ± standard deviation. The * indicates significant difference among treatment groups. ** *P* < 0.01.

**Figure S3. The Pearson correlation coefficient (PCC) analysis of samples in blood (A), liver (B) and adrenal gland (C) tissues.** The PCCs of blood (B), liver (L) and adrenal gland (A) tissues in control (C) and H120 (H) groups were calculated using the FPKM.

**Figure S4.** **Significantly enriched cellular component (CC) and molecular function (MF) terms of blood, liver and adrenal glands in H120 vs. Control comparisons.** (A) All the significantly enriched CC and MF terms of blood. (B and C) The Top 15 significantly enriched CC and MF terms of liver and adrenal gland tissues. The number of up-regulated (red) and down-regulated (blue) DEGs (*Q* < 0.05 and absolute log_2_ (fold-change) > 1) enriched in CC and MF terms in blood, liver and adrenal glands when H120 was compared to control. The x-axis coordinates are represented on a logarithmic scale with a radix of 10.

**Figure S5.** **Significantly enriched biological process (BP) terms of DEGs with** ***Q* < 0.05 and absolute log_2_(fold-change) > 2.32 in liver and adrenal glands in H120 vs. Control comparisons.** (A and B) All the significantly enriched BP of liver (A) and adrenal glands (B). The number of up-regulated (red) and down-regulated (blue) DEGs enriched in BP in liver and adrenal glands when H120 was compared to control. The x-axis coordinates are represented on a logarithmic scale with a radix of 10.

**Figure S6.** **Regression analysis of the significantly differentially expressed gene (DEGs) levels (x-axis) with the biochemical indicators levels of serum (y-axis).** (A) The regression analysis of the expression levels of *Vim*, *S100a4* and *CD14* genes in blood with the biochemical indicators levels in serum (n = 4 per group). (B) The regression analysis of the expression levels of seven genes in the adrenal glands with the biochemical indicators levels in serum (n = 5 per group). The *P* < 0.05 means significant regression. * represents *P* < 0.05 and ** represents *P* < 0.01.
