# Supplementary figures and images for: Heat Stress Impairs the Physiological Responses and Regulates Genes Coding for Extracellular Exosomal Proteins in Rat
Source: Genes (Basel). 2020 Mar 13;11(3):306. doi: 10.3390/genes11030306 (PMC7140893; doi:10.3390/genes11030306)

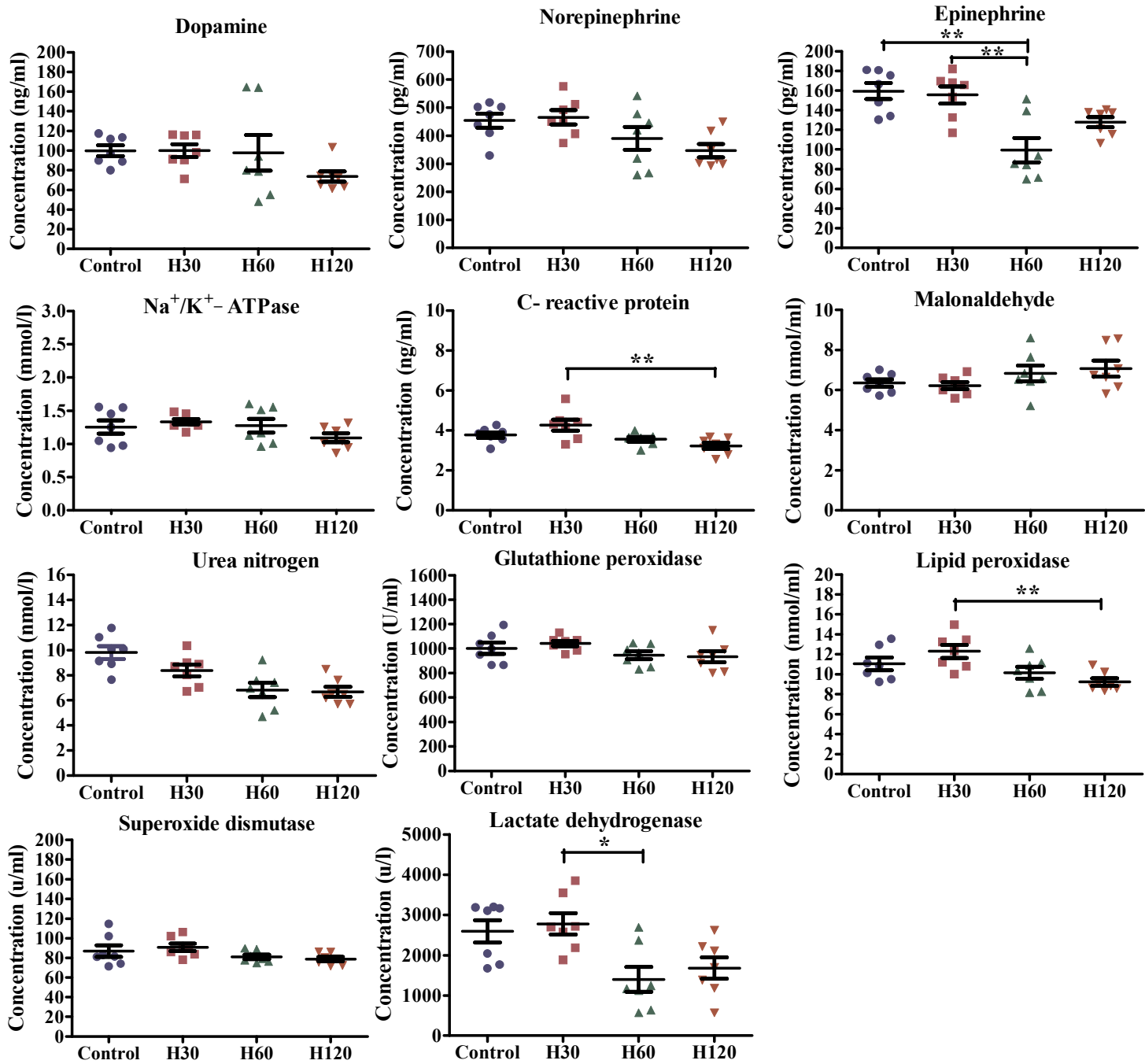

Supplement: Supplementary file 1 [file genes-11-00306-s001.zip › genes-717632-supplementary/Figure S1.pdf]

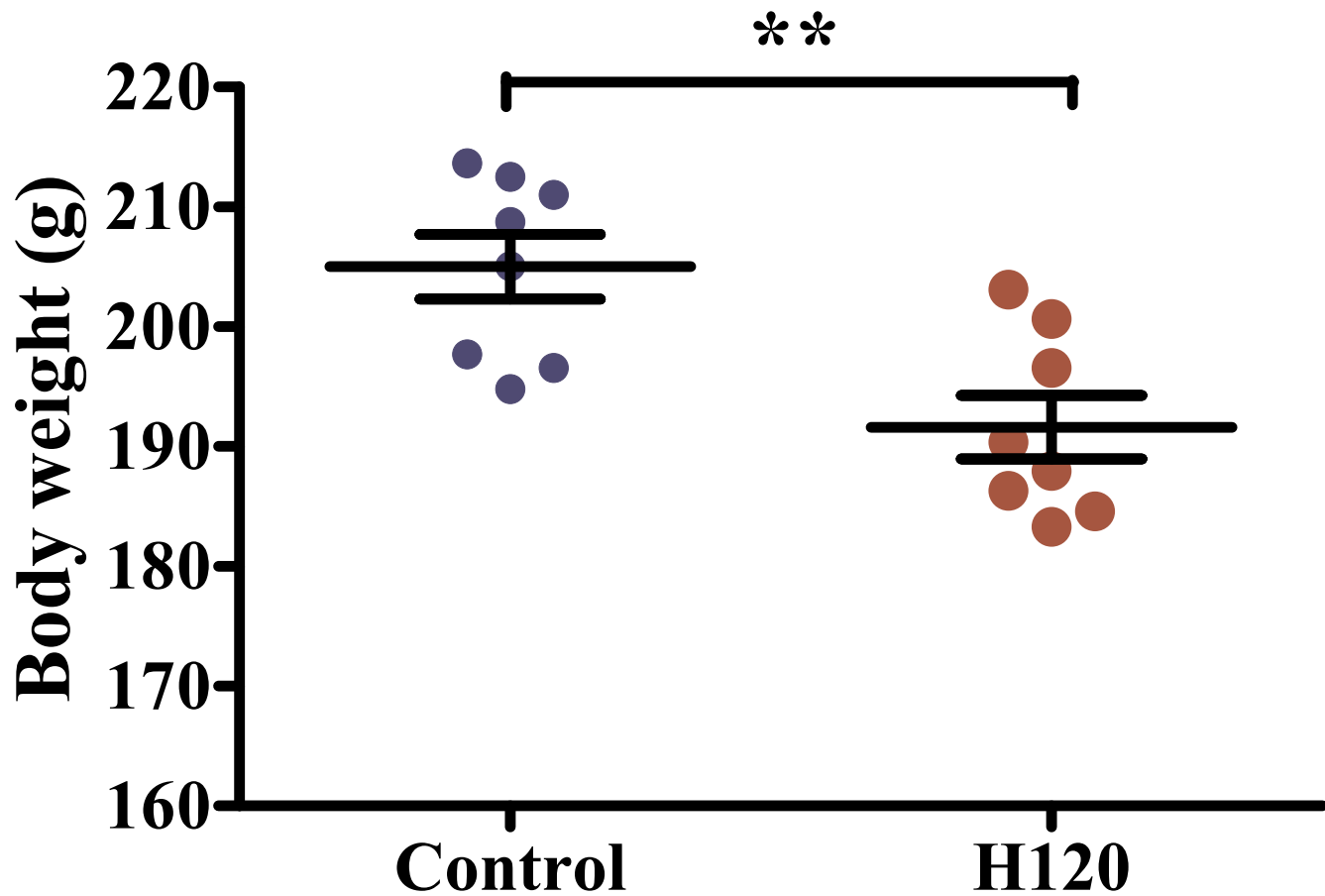

Supplement: Supplementary file 1 [file genes-11-00306-s001.zip › genes-717632-supplementary/Figure S2.pdf]

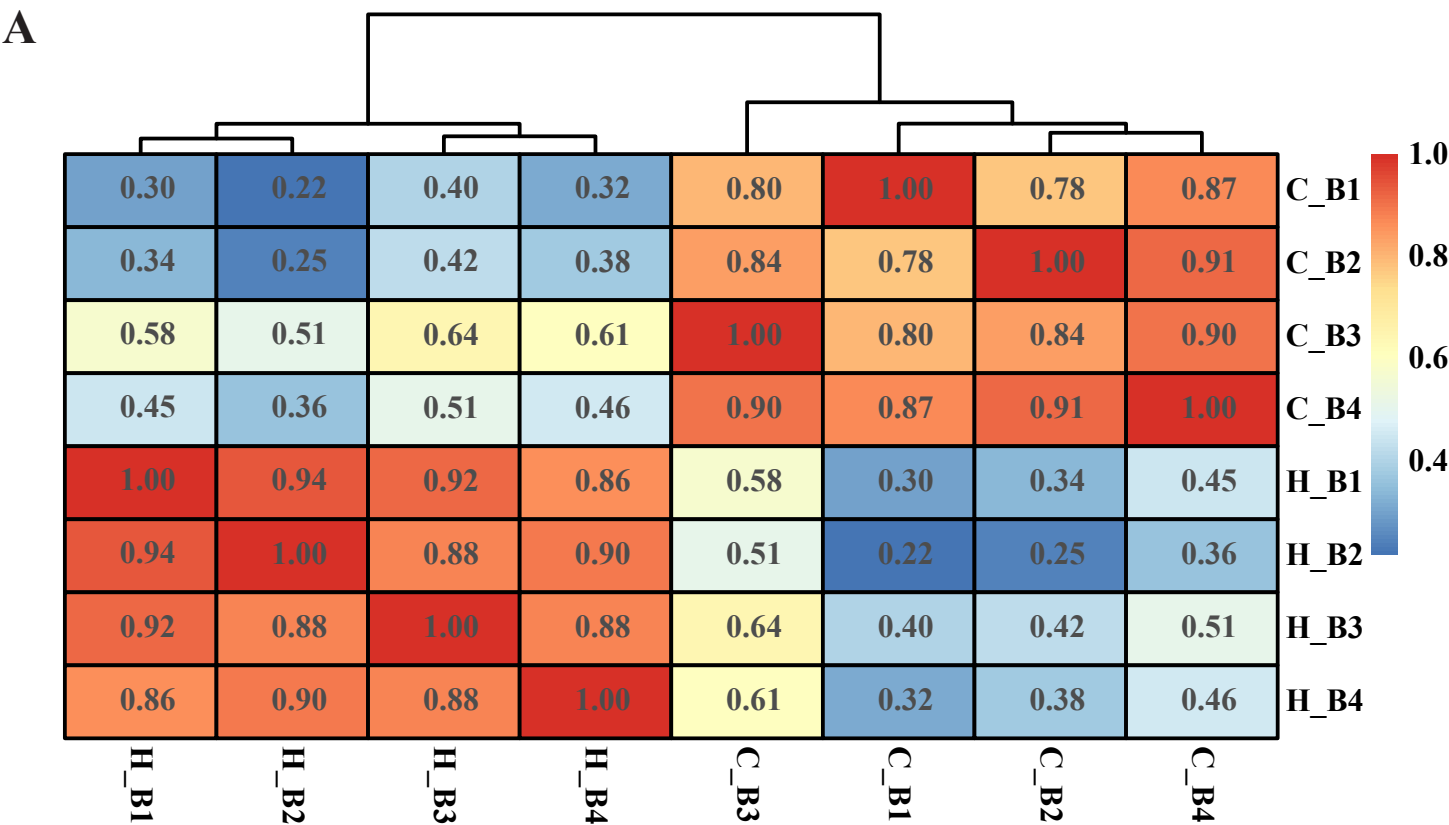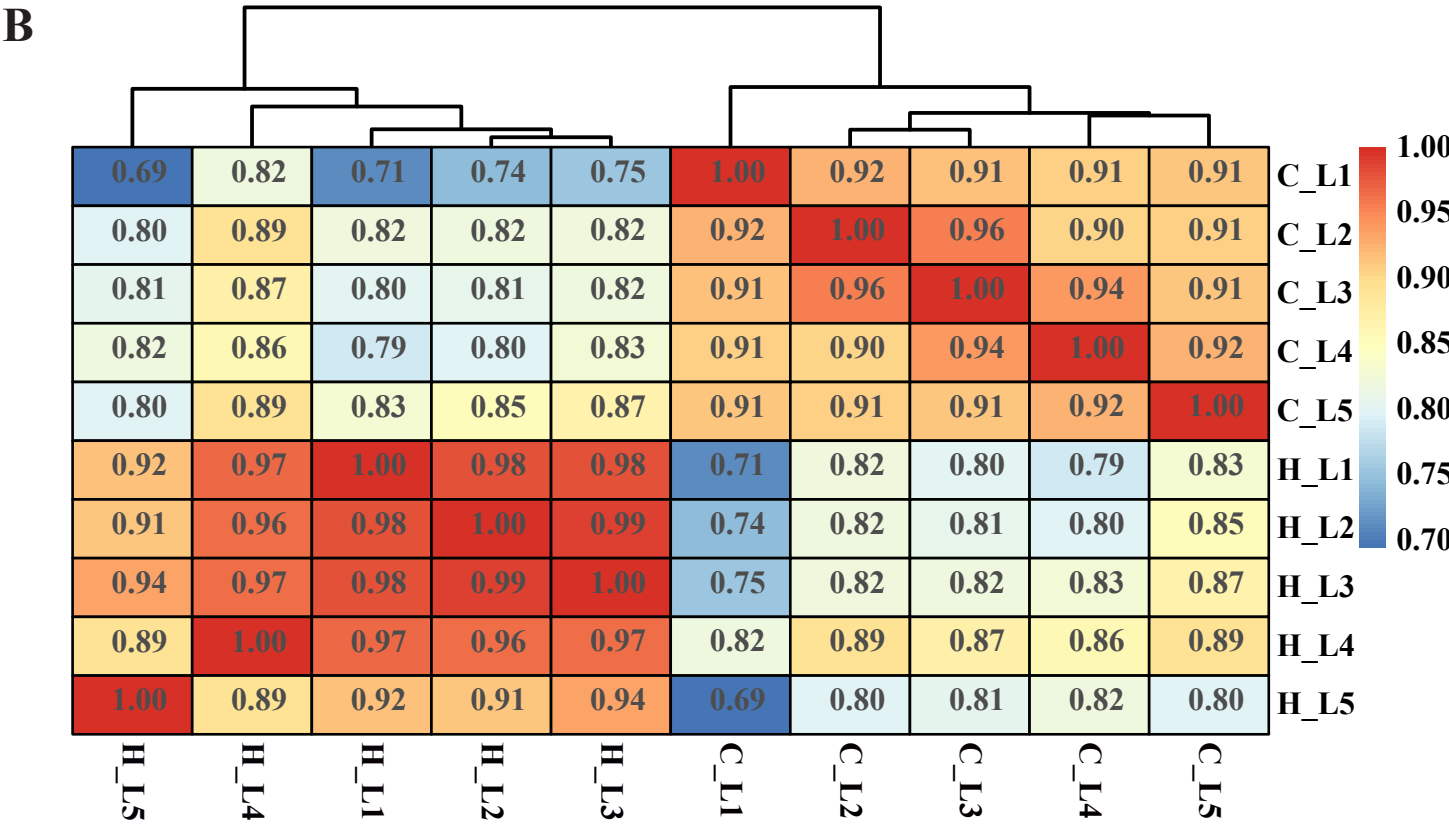

C

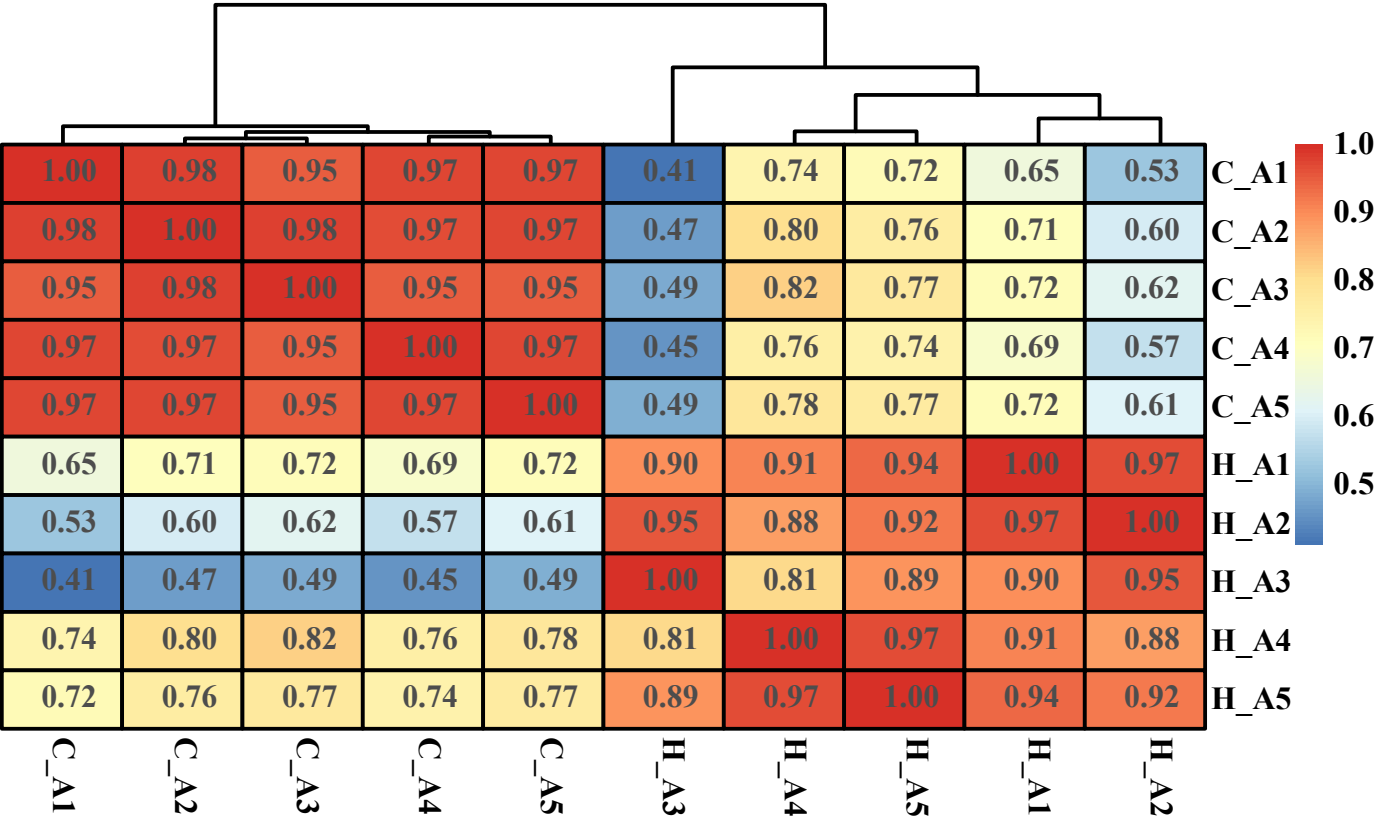

Supplement: Supplementary file 1 [file genes-11-00306-s001.zip › genes-717632-supplementary/Figure S3.pdf]

A

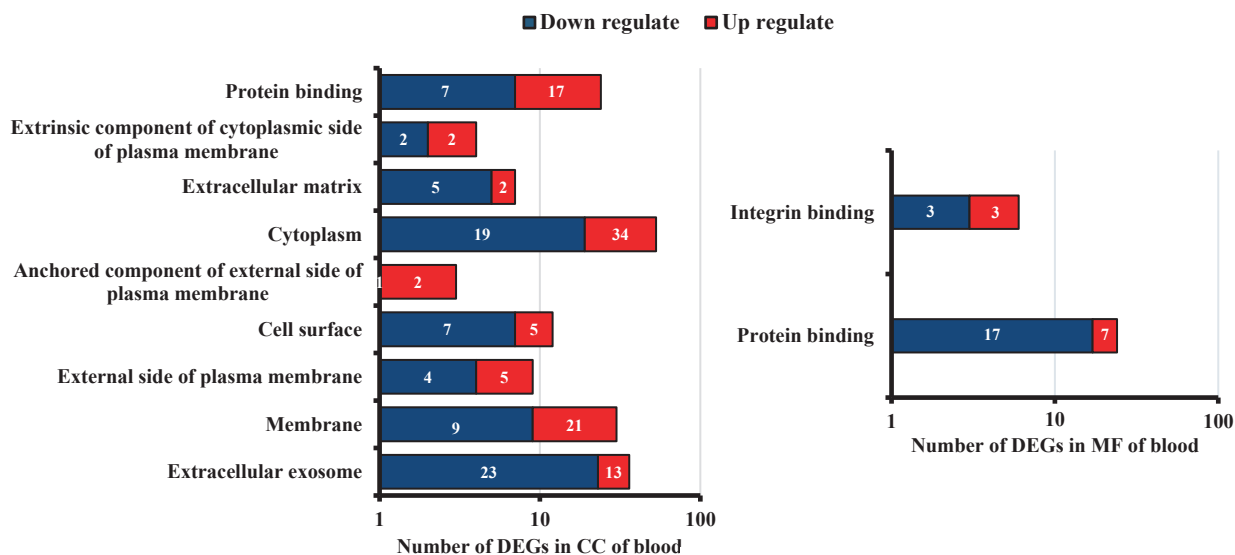

B

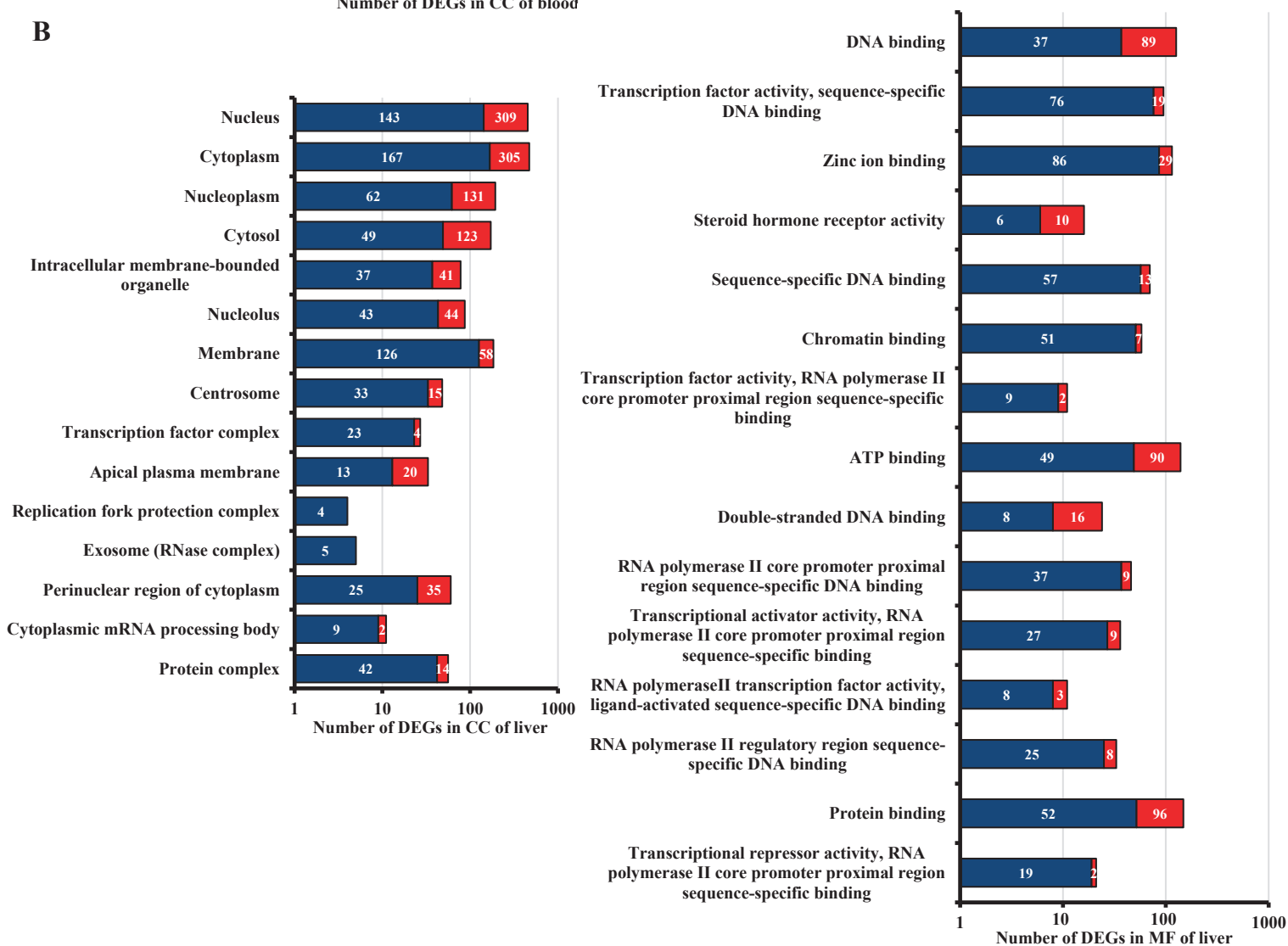

C

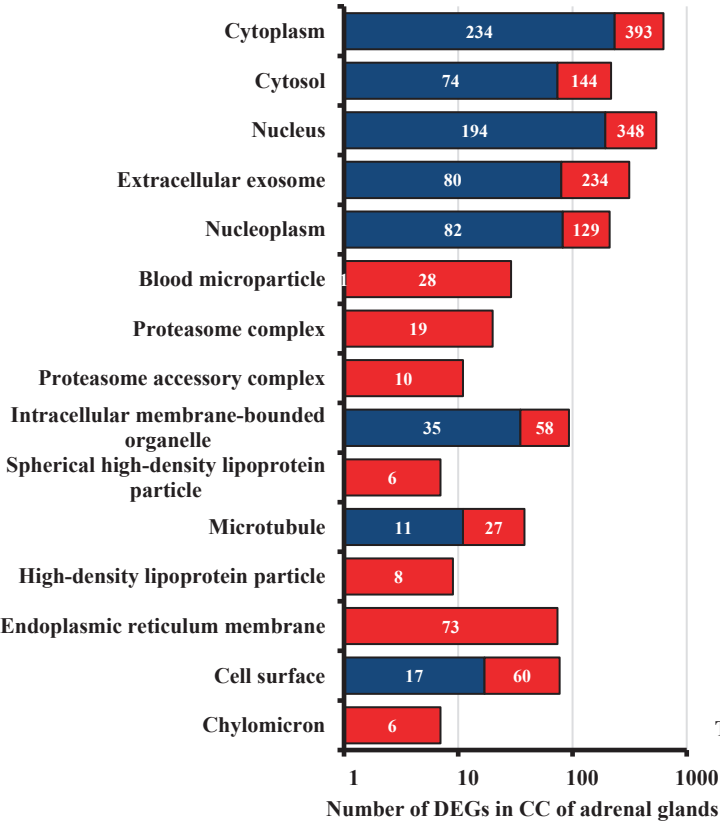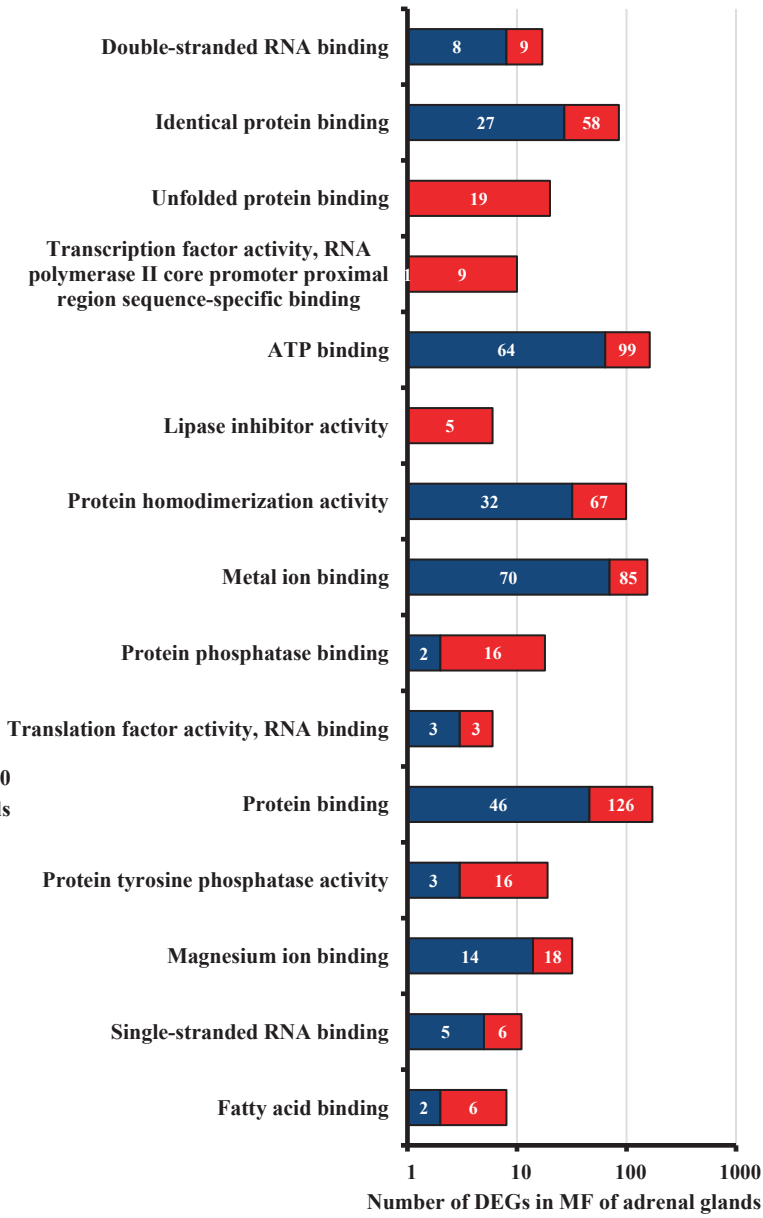

Supplement: Supplementary file 1 [file genes-11-00306-s001.zip › genes-717632-supplementary/Figure S4.pdf]

A

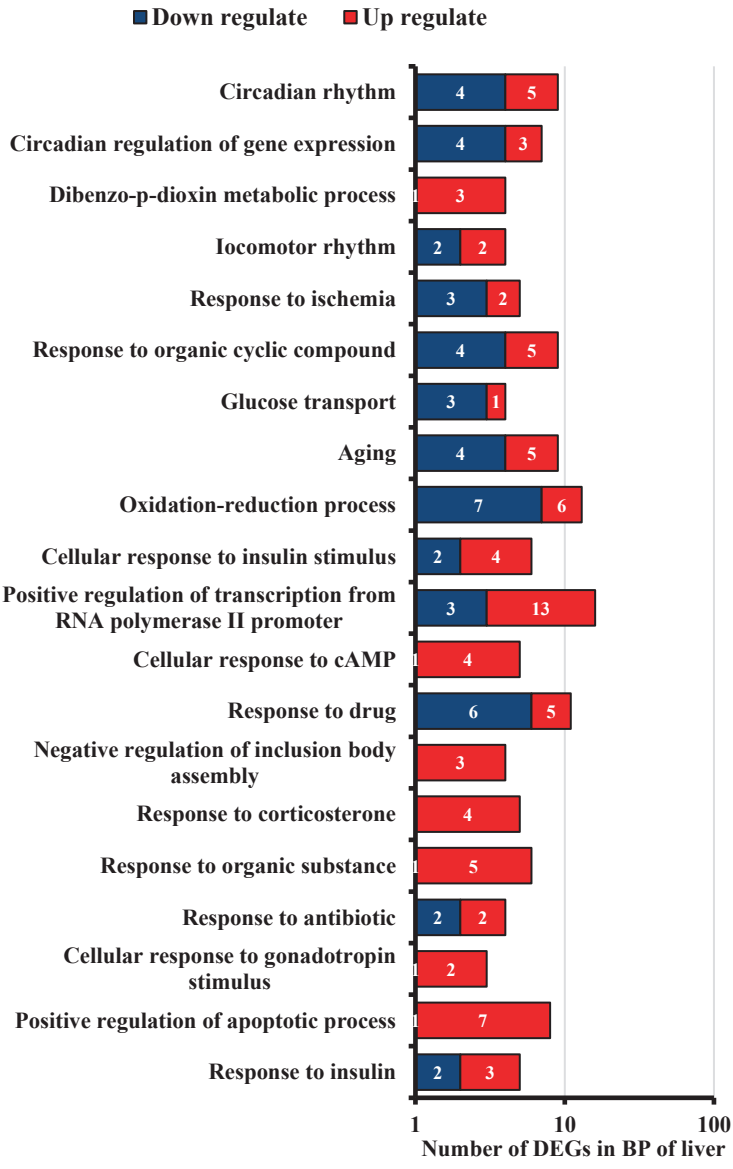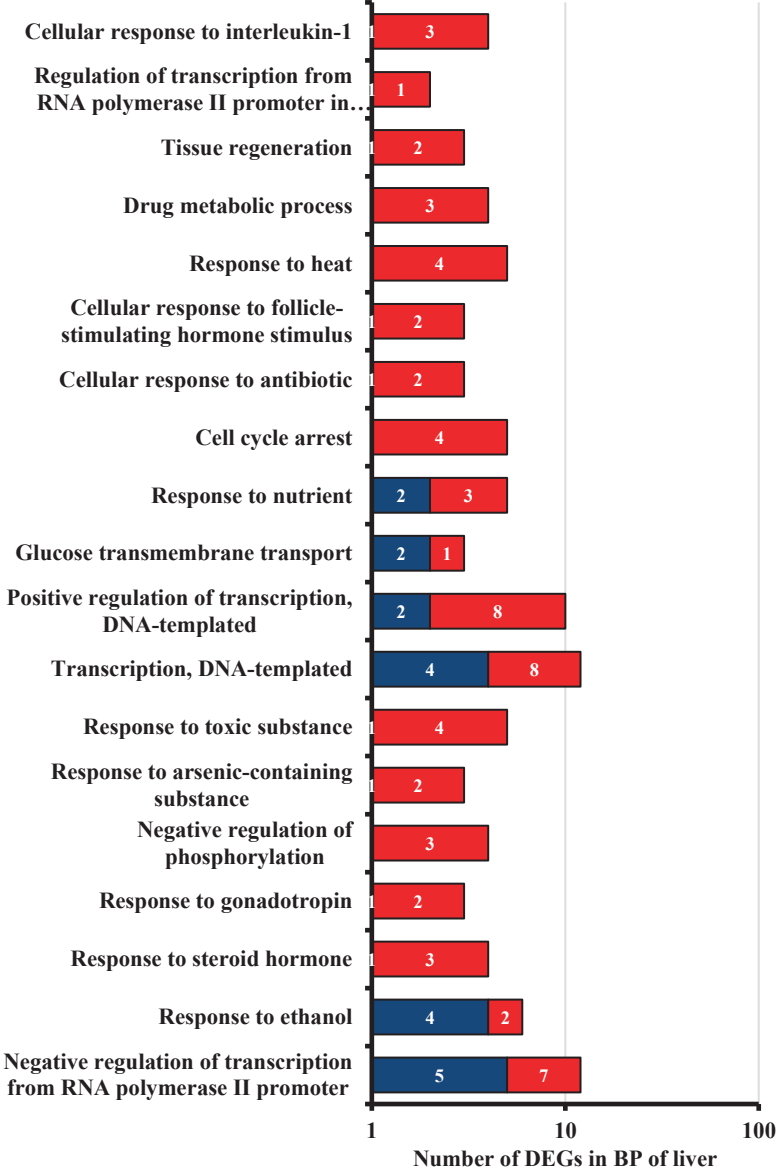

**B**

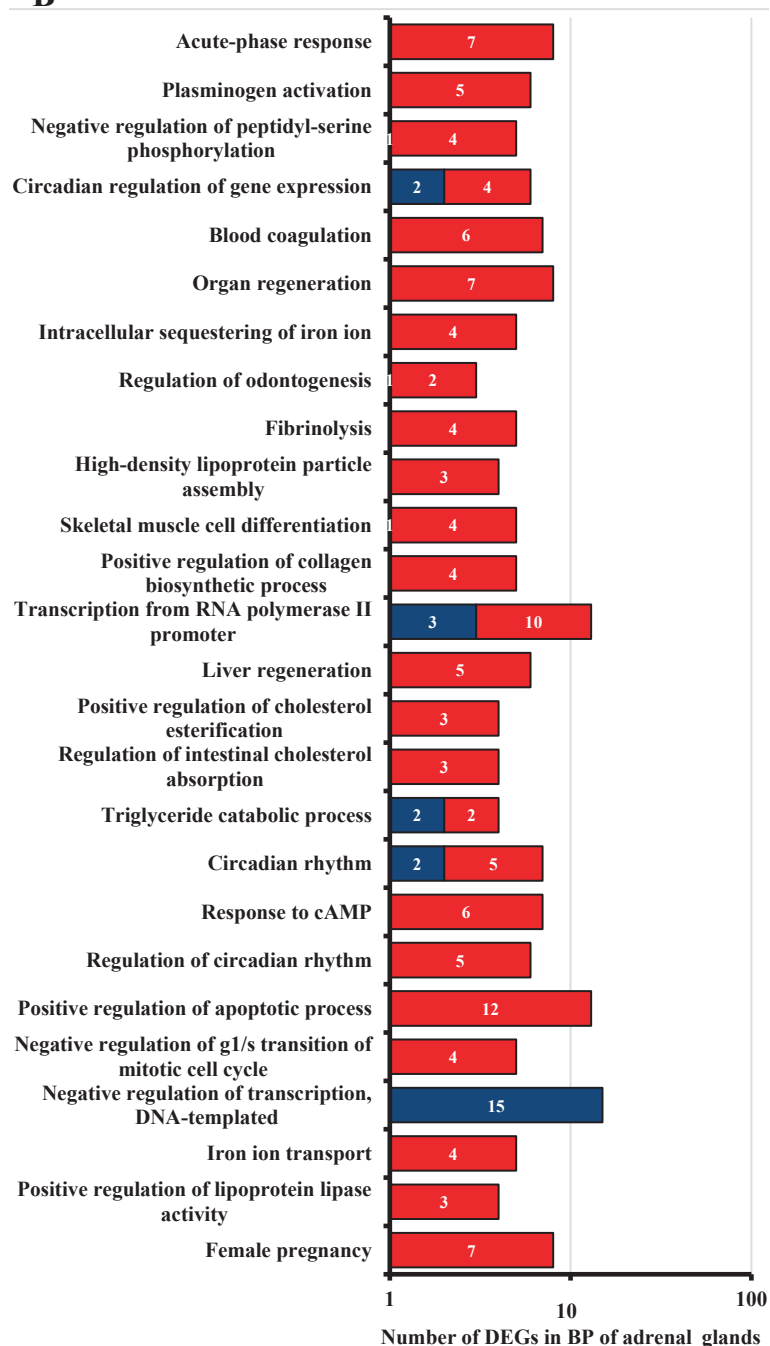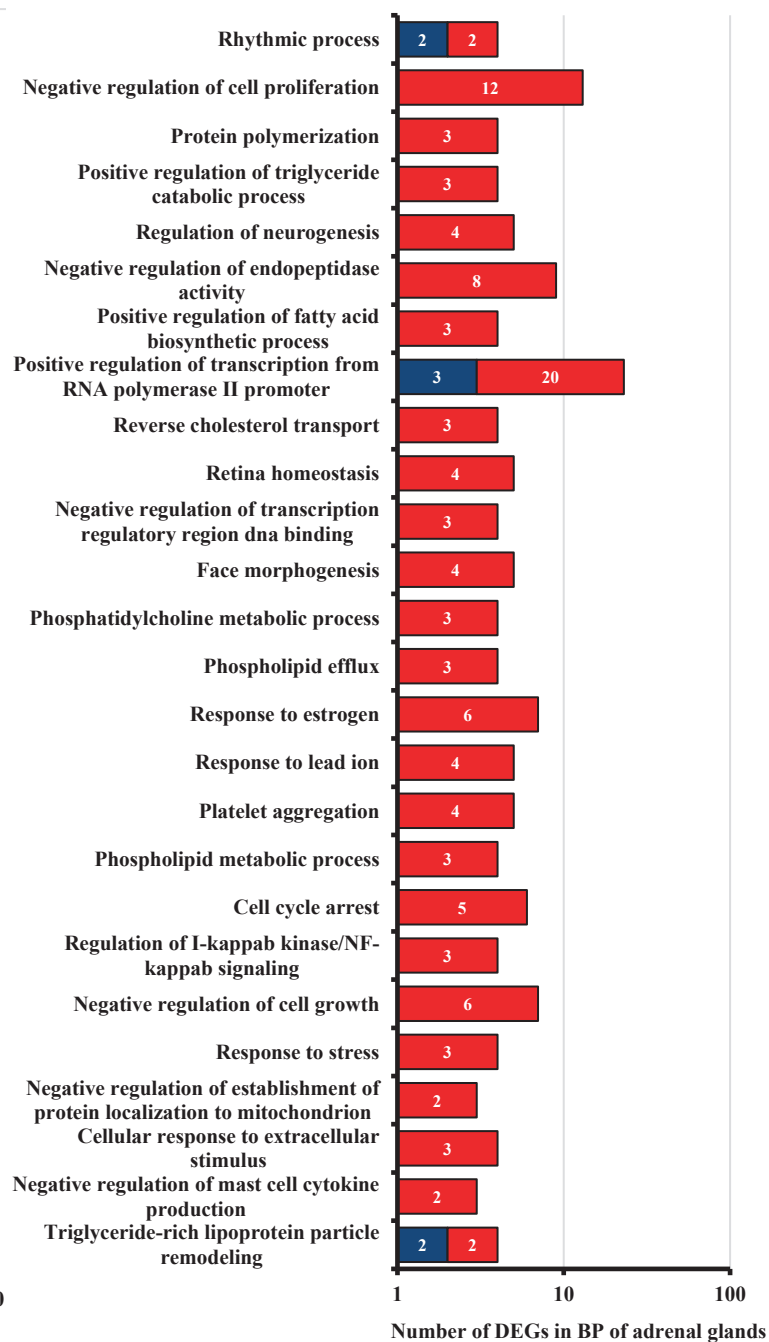

Supplement: Supplementary file 1 [file genes-11-00306-s001.zip › genes-717632-supplementary/Figure S5.pdf]

A

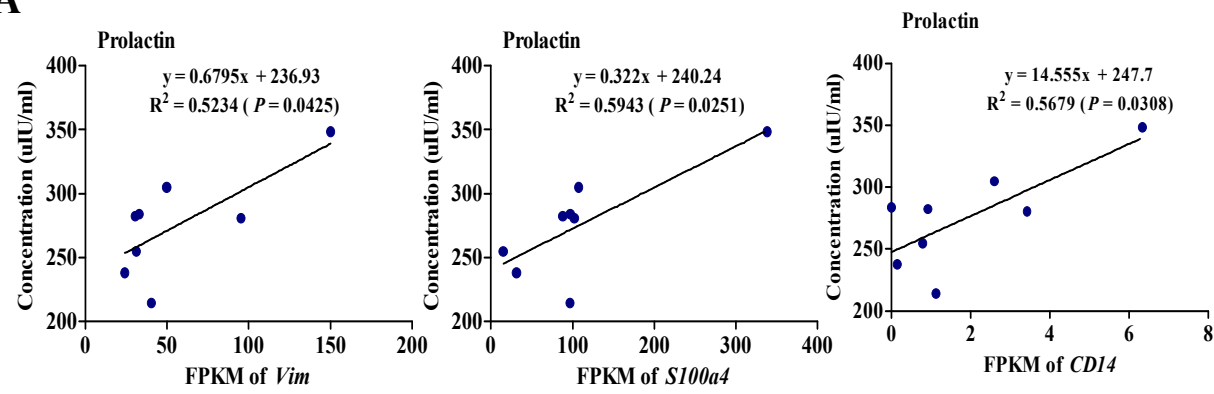

B

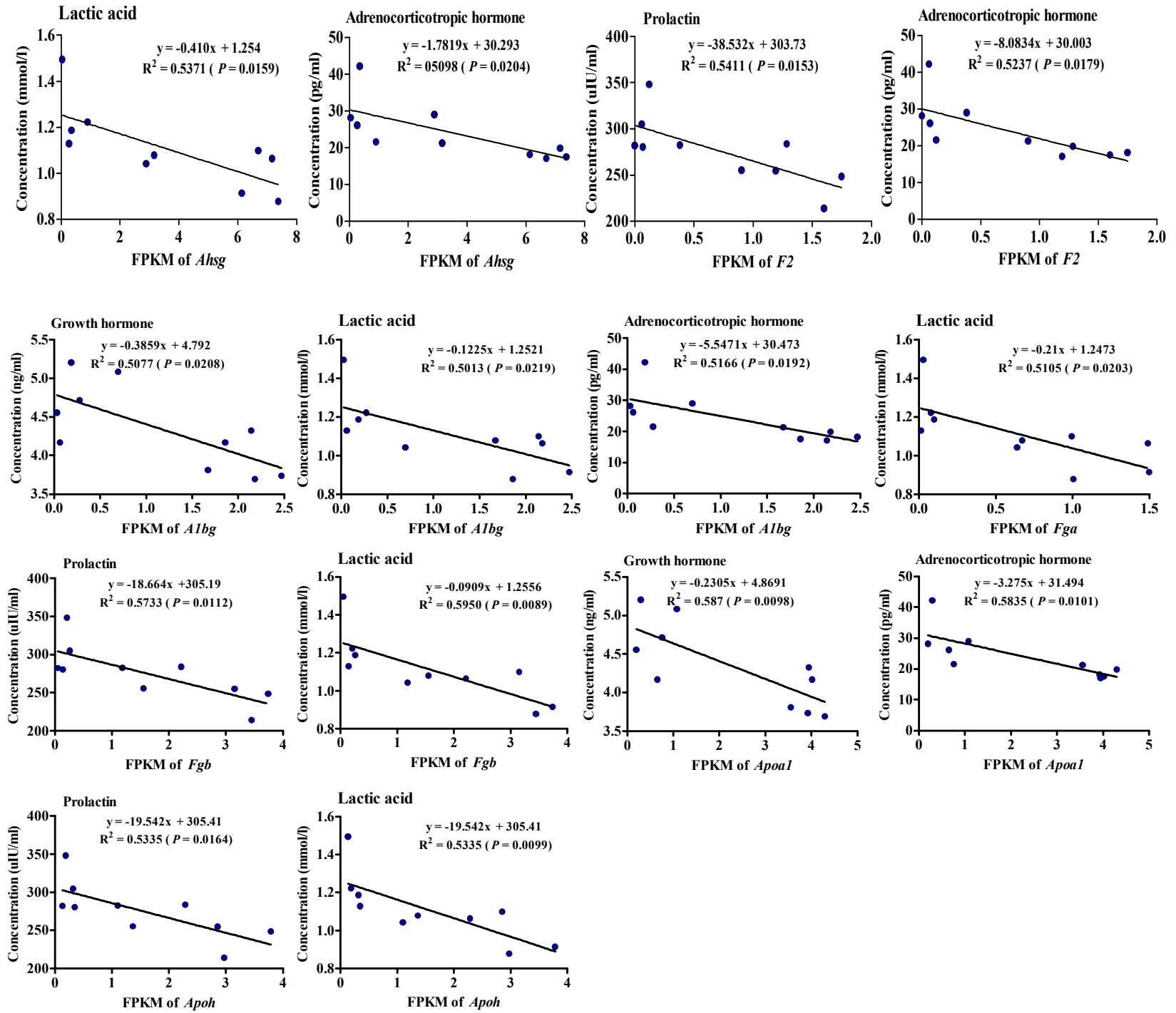

Supplement: Supplementary file 1 [file genes-11-00306-s001.zip › genes-717632-supplementary/Figure S6.pdf]
